# circKDM1A suppresses bladder cancer progression by sponging miR-889-3p/CPEB3 and stabilizing p53 mRNA

**DOI:** 10.1016/j.isci.2024.109624

**Published:** 2024-03-29

**Authors:** Haotian Chen, Jing Wen, Wentao Zhang, Wenchao Ma, Yadong Guo, Liliang Shen, Zhijin Zhang, Fuhan Yang, Yue Zhang, Yaohui Gao, Tianyuan Xu, Yang Yan, Wei Li, Junfeng Zhang, Shiyu Mao, Xudong Yao

**Affiliations:** 1 Department of Urology, Shanghai Tenth People’s Hospital, School of Medicine, Tongji University, Shanghai, China; 2 Urologic Cancer Institute, School of Medicine, Tongji University, Shanghai, China; 3 Institute of Energy Metabolism and Health, Shanghai Tenth People’s Hospital, Tongji University School of Medicine Shanghai, Shanghai 200072, P.R. China; 4 Department of Reproduction, The First Affiliated Hospital of USTC, Division of Life Sciences and Medicine, University of Science and Technology of China, Hefei 230001, China; 5 Department of Urology, The Affiliated People’s Hospital of Ningbo University, Ningbo, China; 6 Department of Central Laboratory, Clinical Medicine Scientific and Technical Innovation Park, Shanghai Tenth People’s Hospital, Shanghai 200435, China; 7 Department of Pathology, Shanghai Tenth People’s Hospital, School of Medicine, Tongji University, Shanghai, China

**Keywords:** Molecular biology, Cell biology, Bioinformatics, Cancer

## Abstract

Circular RNAs (circRNAs) play crucial biological functions in various tumors, including bladder cancer (BCa). However, the roles and underlying molecular mechanisms of circRNAs in the malignant proliferation of BCa are yet unknown. CircKDM1A was observed to be downregulated in BCa tissues and cells. Knockdown of circKDM1A promoted the proliferation of BCa cells and bladder xenograft growth, while the overexpression of circKDM1A exerts the opposite effect. The dual-luciferase reporter assay revealed that circKDM1A was directly bound to miR-889-3p, acting as its molecular sponge to downregulate CPEB3. In turn, the CPEB3 was bound to the CPE signal in p53 mRNA 3′UTR to stabilize its expression. Thus, circKDM1A-mediated CPEB3 downregulation inhibits the stability of p53 mRNA and promotes BCa malignant progression. In conclusion, circKDM1A functions as a tumor suppressor in the malignant proliferation of BCa via the miR-889-3p/CPEB3/p53 axis. CircKDM1A may be a potential prognostic biomarker and therapeutic target of BCa.

## Highlights

• CircKDM1A suppresses BCa malignancy
• CircKDM1A promoted BCa proliferation via the miR-889-3p/CPEB3 axis
• p53 mRNA stability was inhibited by circKDM1A-mediated CPEB3 downregulation

## Graphical abstract

**Figure:**
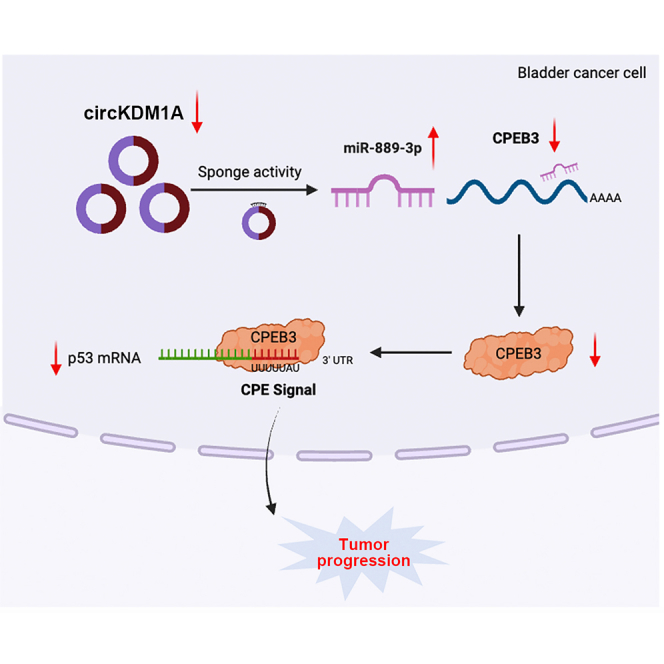

## Introduction

Bladder cancer (BCa) is the second most common urological malignant tumor worldwide, accounting for 549,000 new cases and approximately 200,000 deaths per year.^1^^,^^2^ About 75% of newly diagnosed BCa cases are non-muscle invasive bladder cancer.^3^ Within 5 years, 10%–30% of patients with the non-invasive disease progress to muscle-invasive bladder cancer (MIBC), characterized by higher rates of metastasis and a poorer prognosis.^4^ Although immunotherapy is widely used for advanced BCa, only 10% of patients benefit. Currently, there are still significant challenges in the treatment of advanced BCa. There are still no clear diagnostic biomarkers available for detecting BCa recurrence. This is also a gap faced by both clinical and basic research.

Circular RNAs (circRNAs) are a class of non-coding RNAs that lack a 5′ methylguanosine cap and a 3′ poly-A tail, allowing them to fold into a single-stranded, covalently closed circular structure. Overwhelming evidence showed that circRNAs perform decisive biological functions and were involved in the tumorigenesis and development of various cancers.^5^^,^^6^ These transcripts are located in various parts of the cell and influence tumor biological behavior through multiple mechanisms. CircRNAs in the nucleus primarily participate in regulating transcription, alternative splicing, and chromatin circularization. They can pause or terminate transcription by forming RNA-DNA hybrids.^7^ Additionally, they act as protein scaffolds, promoting the colocalization of enzymes and their substrates.^8^ CircRNAs located in the cytoplasm typically function as competing endogenous RNAs (ceRNAs), absorbing miRNAs through sponges and subsequently affecting targeted mRNAs.^9^ They can act as protein decoys, scaffolds, and recruiters to facilitate or disrupt protein interactions in various physiological and pathological contexts.^10^ In addition, some circRNAs can also play a role in protein translation.^11^ Our previous research discovered that hsa_circ_0004296 inhibits prostate cancer metastasis by interacting with EIF4A3 to prevent the nuclear export of ETS1 mRNA.^12^ Although circRNAs have been explored in certain cancers, the expression, clinical significance, and molecular mechanisms of newly identified circRNAs in BCa remain uninvestigated.

Cytoplasmic polyadenylation elements (CPEBs) bind to CPE in the 3′ untranslated region (UTR) of mRNA and are a key factor in controlling polyA tail elongation and polyadenylation-induced translation.^13^ CPEBs contain several binding sites for microRNAs (miRNAs), and their function is regulated by corresponding miRNAs. Studies have demonstrated that CPEB3 may act as a tumor suppressor in regulating the proliferation, invasion, and apoptosis of various tumors.^14^^,^^15^ The p53 is a frequently mutated tumor suppressor gene in approximately 50% of tumors. Various stress signals, including DNA damage, oncogene activation, hypoxia, and ribosome stress, can activate p53.^16^ Activated p53 can perform its tumor suppressor function through several mechanisms, such as promoting apoptosis, trapping the cell cycle, repairing DNA damage, regulating metabolism, and inducing ferroptosis.^17^ Although there are many reports related to p53, the mechanism by which *CPEB3* regulates p53 has not yet been elucidated in BCa.

In this study, we identified a novel tumor suppressor circRNA called circKDM1A (hsa_circ_0009061) using a circRNA microarray. This molecule is derived from exons 3, 4, and 5 of the lysine demethylase 1A (KDM1A) gene and is significantly downregulated in BCa. We showed its low expression is associated with BCa malignant progression *in vitro* and *in vivo*. We also demonstrated that circKDM1A sponges miR-889-3p to regulate the CPEB3/p53 axis. Our study implies that circKDM1A is a potential diagnostic biomarker and therapeutic target in BCa.

## Results

### Low expression of circRNA known as lysine demethylase 1A is related to poor prognosis of bladder cancer

**Figure 1:**
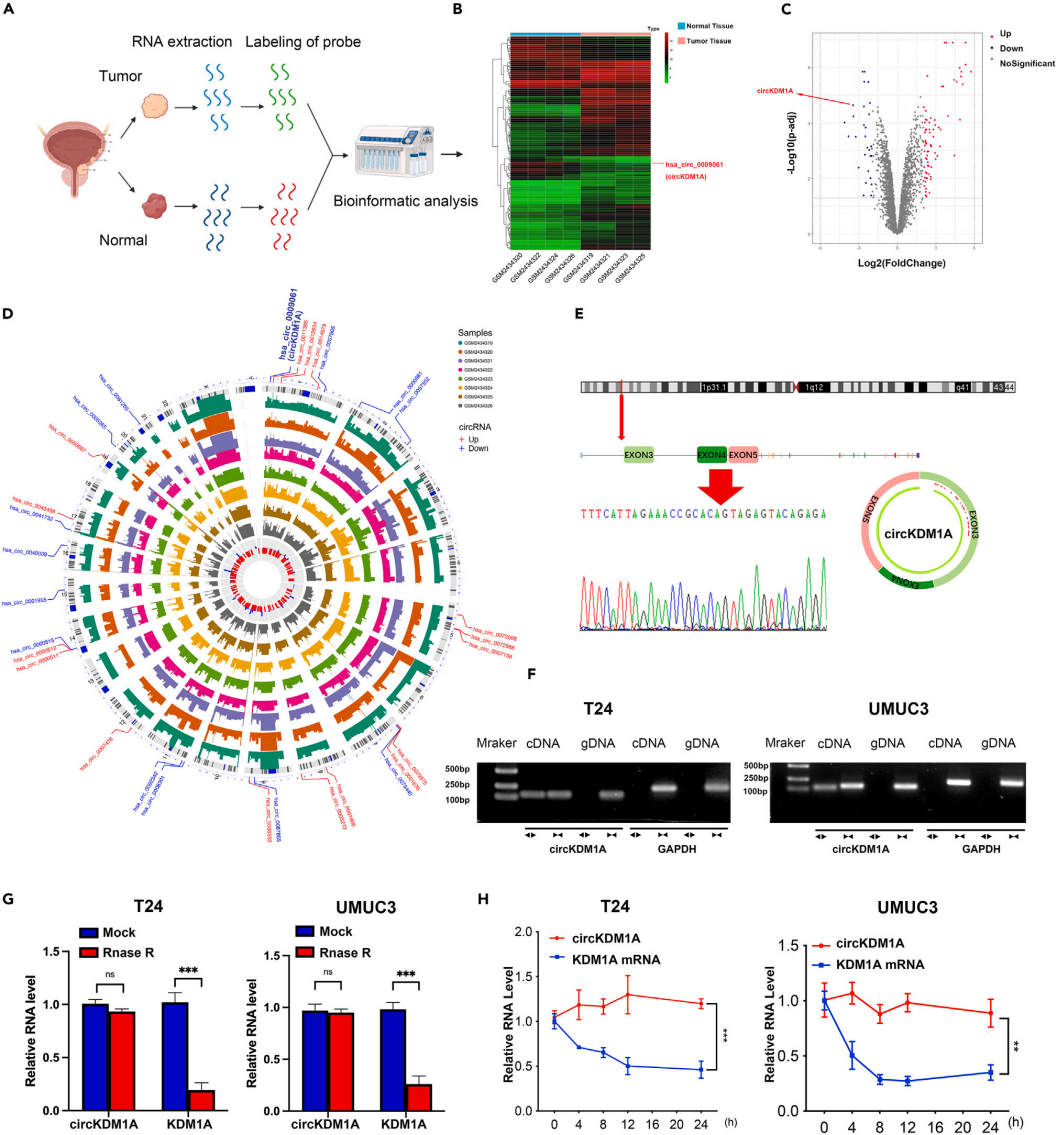
CircRNAs expression profiles in BCa and characterization of circKDM1A (A) The process of circRNAs microarray. (B and C) The heatmap and volcano plot of differentially expressed circRNAs. (D) The expression levels and chromosome positions of the differentially expressed circRNAs. (E) The schematic diagram of the genomic location and splicing pattern of circKDM1A. (F) Gel electrophoresis showed that circKDM1A could be detected only in cDNA and not in gDNA. (G and H) RNase-R and actinomycin D treatment assays showed no change in the expression level of circKDM1A, while the expression of KDM1A mRNA was significantly decreased. The data were presented as the mean ± SEM obtained from at least three independent experiments. Statistical significance in (G) and (H) was determined by Student’s t test. ∗p < 0.05, ∗∗p < 0.01, ∗∗∗p < 0.001.

CircRNAs are involved in the malignant progression of many cancers, but whether they have a similar role in BCa is unknown. Therefore, we analyzed 4 pairs of human BCa and matched para-carcinoma tissues using the GSE92675 circRNA microarray dataset to find differentially expressed circRNAs (Figure 1A). A total of 107 differentially expressed circRNAs were identified (p < 0.05) with 79 upregulated and 28 downregulated (Figures 1B and 1C). The chromosomal locations of the top 30 differentially expressed circRNAs are displayed in Figure 1D. Next, we designed divergent primers to detect 5 screened circRNA candidates in BCa cell lines, and baldder normal epithelial cells. We chose hsa_circ_0009061 (chr1:23356961-23377013) as the candidate circRNA for further research. This circRNA known as lysine demethylase 1A (circKDM1A) was formed through the back-splicing of exons 3, 4, and 5 of the KDM1A gene. The reasons were as followings: First, the circKDM1A that are smaller than 2000 bp and annotated by the circBase database is selected. Then, compared to that in bladder normal cells, circKDM1A expression was significantly downregulated in all BCa cell lines (Figures S1A–S1D).We designed primers specific to the circular site of circKDM1A to confirm its stable circular structure and confirmed the back-spliced junction of circKDM1A by Sanger sequencing (Figure 1E). The presence of circKDM1A in T24 and UMUC3 was verified by gel electrophoresis (Figure 1F). We also validated circKDM1A stability using qPCR after treating the transcript with RNase R and actinomycin D, showing that circKDM1A has better stability than linear mRNA (Figures 1G and 1H).

### Knocking down circRNA known as lysine demethylase 1A promotes bladder cancer proliferation *in vivo*

**Figure 2:**
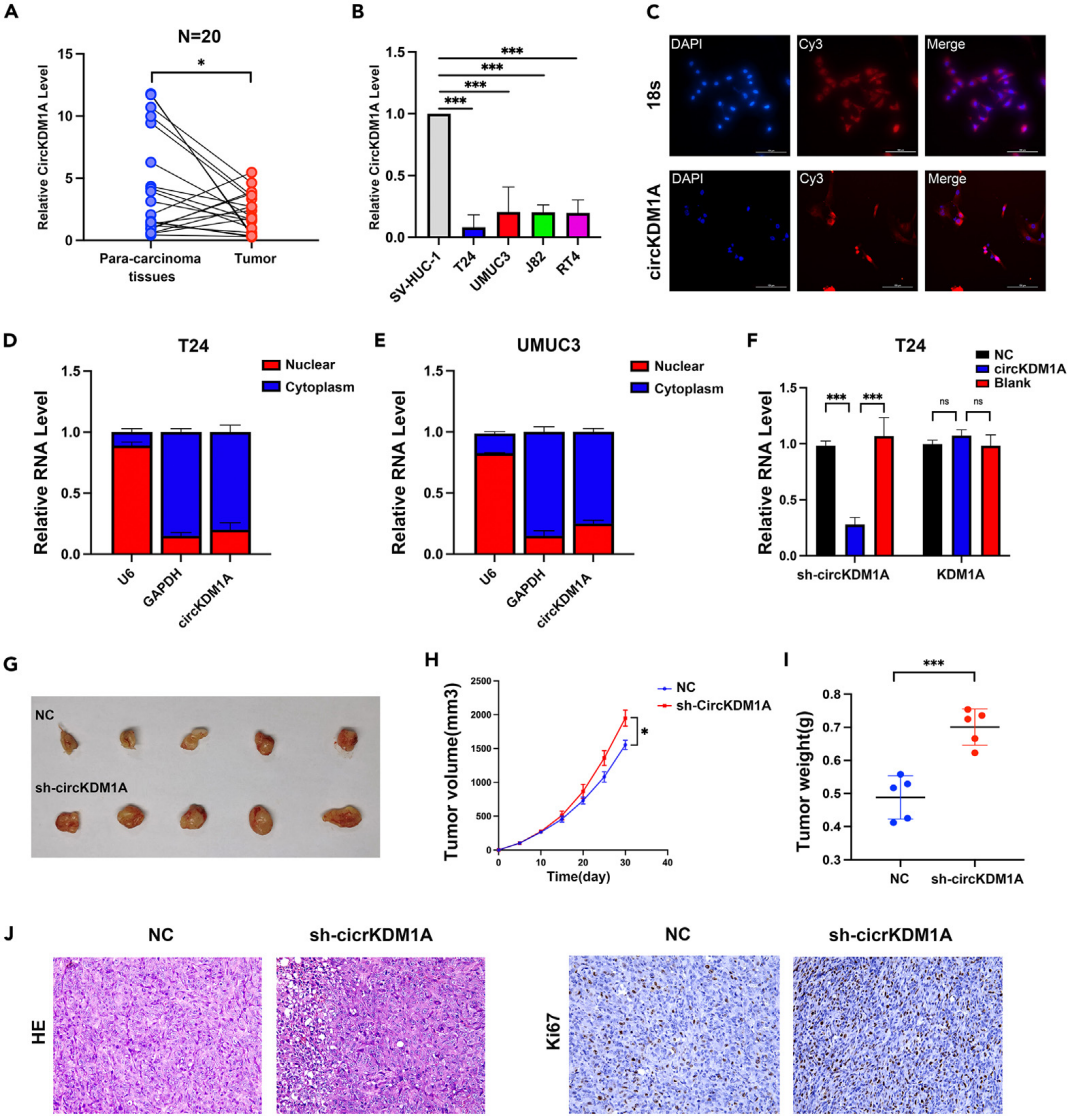
Localization of circKDM1A in cells and establishment of the sh-circKDM1A mouse model (A) The expression of circKDM1A in tumors and para-carcinoma tissues of our clinical data (n = 20). (B) CircKDM1A was significantly low-expressed in various BCa cell lines. (C) FISH images revealed that circKDM1A was expressed in both the cytoplasm and nucleus. (D and E) Cytoplasmic and nuclear mRNA fractionation experiment showed that circKDM1A is mainly located in the cytoplasm. (F) Validation of circKDM1A knockdown efficiency by qPCR. (G–I) Nude mice were injected with NC and circKDM1A T24 cell line, and analysis of tumor specimens (G), tumor volume (H), and tumor weight (I) in the mice bearing subcutaneous xenografts were performed. (J) HE staining of subcutaneous xenografts tumors in nude mice and histochemical staining of proliferation marker Ki67.The data were presented as the mean ± SEM obtained from at least three independent experiments. Statistical significance was determined using either Student’s t test or one-way ANOVA as appropriate. ∗p < 0.05, ∗∗p < 0.01, ∗∗∗p < 0.001.

We used 20 BCa and para-carcinoma tissues to verify circKDM1A expression by qPCR. We showed that circKDM1A levels were highly elevated in para-carcinoma tissues versus the BCa (Figure 2A). Moreover, circKDM1A was significantly downregulated in T24, UMUC3, J82, and RT-4 tumor cell lines, compared with the normal urothelial cell line SV-HUC-1 (Figure 2B). Subsequent nuclear-plasma extraction and RNA-FISH assays demonstrated that circKDM1A has a predominantly cytoplasmic localization (Figures 2C–2E). We used plasmids to construct stable circKDM1A knockdown and overexpression T24 and UMUC3 cell lines and verified the knockdown and overexpression efficiency by qPCR (Figure 2F; Figure S2A). Next, we constructed a subcutaneous xenograft mouse model using the circKDM1A knockdown T24 cell line to investigate whether circKDM1A affects BCa proliferation *in vivo*. Indeed, knocking down circKDM1A promoted BCa tumor growth and substantially increased tumor volume and weight in the tumor-bearing mice compared with the mice in the NC group (Figures 2G–2I). Using IHC, we found that the mice in the knockdown circKDM1A group exhibited the enhanced expression of the tumor proliferation marker antigen Ki67 compared to those in the NC group (Figure 2J).

### Overexpressing circRNA known as lysine demethylase 1A suppresses proliferation and promotes apoptosis in bladder cancer *in vitro*

**Figure 3:**
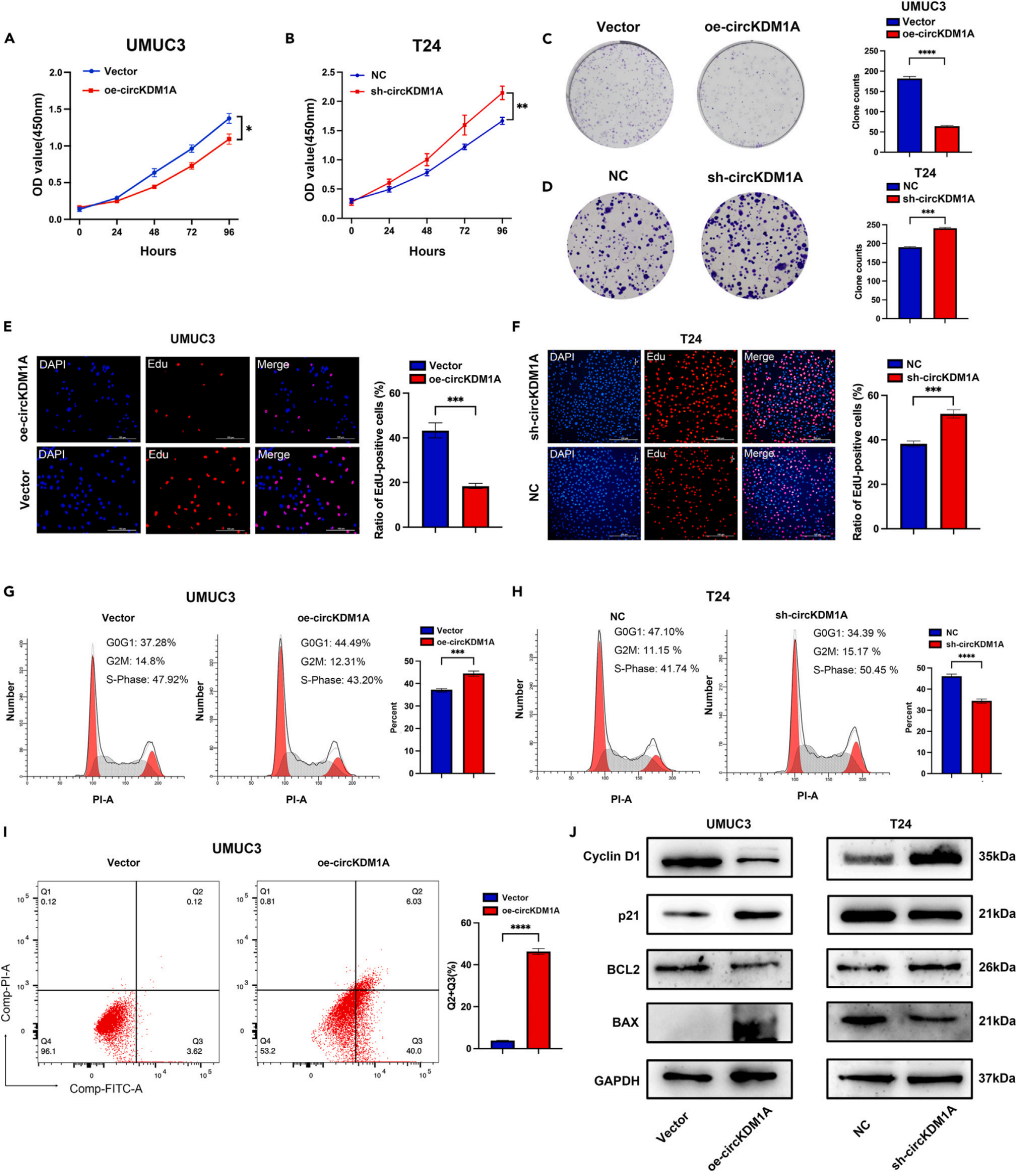
CircKDM1A can affect BCa cell proliferation and apoptosis *in vitro* (A) CCK-8 assay showed that the overexpression of circKDM1A inhibited cell proliferation in UMUC3. (B) CCK-8 assay showed that the knockdown circKDM1A increased cell proliferation in T24. (C) The proliferation of BCa cells transfected with overexpressing circKDM1A was assessed using the clone formation assay in UMUC3. (D) The proliferation of BCa cells transfected with knockdown circKDM1A was assessed using the clone formation assay in T24. (E) EdU assay showed that the overexpression of circKDM1A inhibited cell proliferation in UMUC3. (F) EdU assay showed that knockdown circKDM1A promoted cell proliferation in T24. (G and H) Cell cycle analysis showed that the overexpression of circKDM1A can cause cells to be arrested in the G0G1 phase (G), while knocking down circKDM1A eliminates this arrest (H). (I) Flow cytometric apoptosis analysis shows that the overexpression of circKDM1A can significantly increase cell apoptosis. (J) Western blot showed that proliferation-related proteins (p21 and cyclinD1) and apoptosis-related proteins (BCL2 and BAX) were significantly changed after knocking down or overexpressing circKDM1A.The data were presented as the mean ± SEM obtained from at least three independent experiments. Statistical significance was determined using Student’s t test. ∗p < 0.05, ∗∗p < 0.01, ∗∗∗p < 0.001, ∗∗∗∗p < 0.0001.

We verified cirKDM1A functions on tumor growth *in vitro* using CCK-8, colony formation, and EdU assays. We showed that circKDM1A overexpression suppressed the proliferation ability of UMUC3 cells (Figures 3A–3C and 3E). Conversely, circKDM1A knockdown induced the proliferation ability of T24 and UMUC3 cells (Figures 3B–3D and 3F; Figures S2B–S2D).

We also uncovered with flow cytometry that overexpressing circKDM1A considerably lengthened the G0G1 phase of the cell cycle, whereas knocking down circKDM1A had the opposite effect (Figures 3G and 3H). In addition, our flow cytometry apoptosis analysis showed that overexpressing circKDM1A increased the proportion of cells undergoing apoptosis (Figure 3I). Quantifying the levels of cell cycle-related proteins (p21 and Cyclin D1) and apoptosis-related proteins (Bax and Bcl2) with Western blotting revealed that circKDM1A overexpression and knockdown had an opposing effect on these proteins (Figure 3J).

### CircRNA known as lysine demethylase 1A regulates bladder cancer progression via miR-889-3p sponge

We further investigated the mechanism by which circKDM1A affects BCa progression. Evidence suggests cytoplasmic circRNAs often bind to miRNAs to function as their molecular sponges and regulate gene expression.

**Figure 4:**
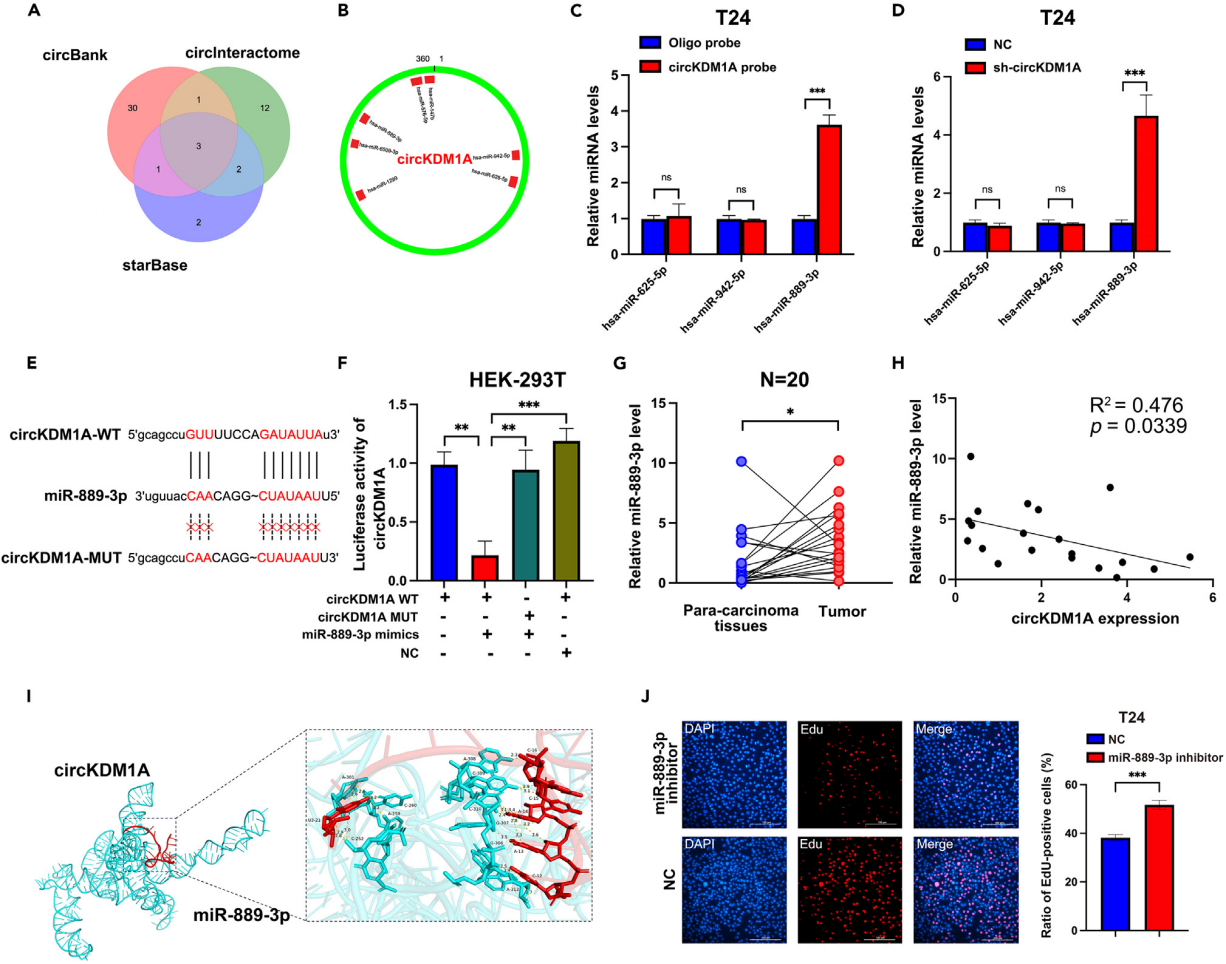
CircKDM1A regulates BCa progression via miR-889-3p sponge (A) Construct a Venn diagram through multiple circRNA databases to predict the miRNAs that circKDM1A may bind to. (B) Schematic drawing showing the potential miRNAs that might bind circKDM1A. (C) miR-889-3p can directly bind to circKDM1A by RNA pull down experiments. (D) qPCR showed that the expression of miR-889-3p was significantly increased after knocking down circKDM1A in T24 cell line. (E) Schematic of circKDM1A wild-type (WT) and mutant (Mut) luciferase reporter vectors. (F) Dual-luciferase reporter demonstrates that miR-889-3p binds to circKDM1A. (G and H) qPCR demonstrates that the miR-889-3p level are significantly high in tumor tissues (G) and negatively correlated with circKDM1A (H) (n = 20). (I) PyMOL software displays the interaction between circKDM1A and miR-889-3p in their 3D structures. (J) EdU experiments showed that miR-889-3p inhibitor suppressed the proliferation of BCa cells.The data were presented as the mean ± SEM obtained from at least three independent experiments. Statistical significance was determined using either Student’s t test or one-way ANOVA as appropriate. ∗p < 0.05, ∗∗p < 0.01, ∗∗∗p < 0.001.

Starbase, CircInteractome, and circBank were used to predict miRNA binding. Seven miRNAs were identified, with three presents in all three databases (hsa-miR-625-5p, hsa-miR-889-3p, hsa-miR-942-5p; Figures 4A and 4B). Subsequently, the RNA pull down by biotinylated probes were performed on three candidate miRNAs. The results showed that only miR-889-3p could be abundantly pulled down by the circKDM1A probe (Figure 4C, p < 0.001). We confirmed with qPCR that knocking down circKDM1A upregulates the expression of miR-889-3p (Figure 4D). Moreover, the luciferase activities of the circKDM1A wild-type reporter were significantly reduced when transfected with miR-889-3p mimics compared with the mutated reporter (Figures 4E and 4F), demonstrating circKDM1A binds and represses miR-889-3p *in vitro*. In addition, miR-889-3p expression significantly increased in the tumor samples and showed a negative correlation with circKDM1A (Figures 4G and 4H). We also assessed the 3D structures of circKDM1A and miR-889-3p with the PyMOL software (Figure 4I; Figure S3A). In addition, the EdU assay showed that the inhibition of miR-889-3p reduced the proliferation of BCa cells compared to the NC group (Figure 4J; Figures S3B and S3C).

### Cytoplasmic polyadenylation element 3 functions as the target of miR-889-3p in bladder cancer

**Figure 5:**
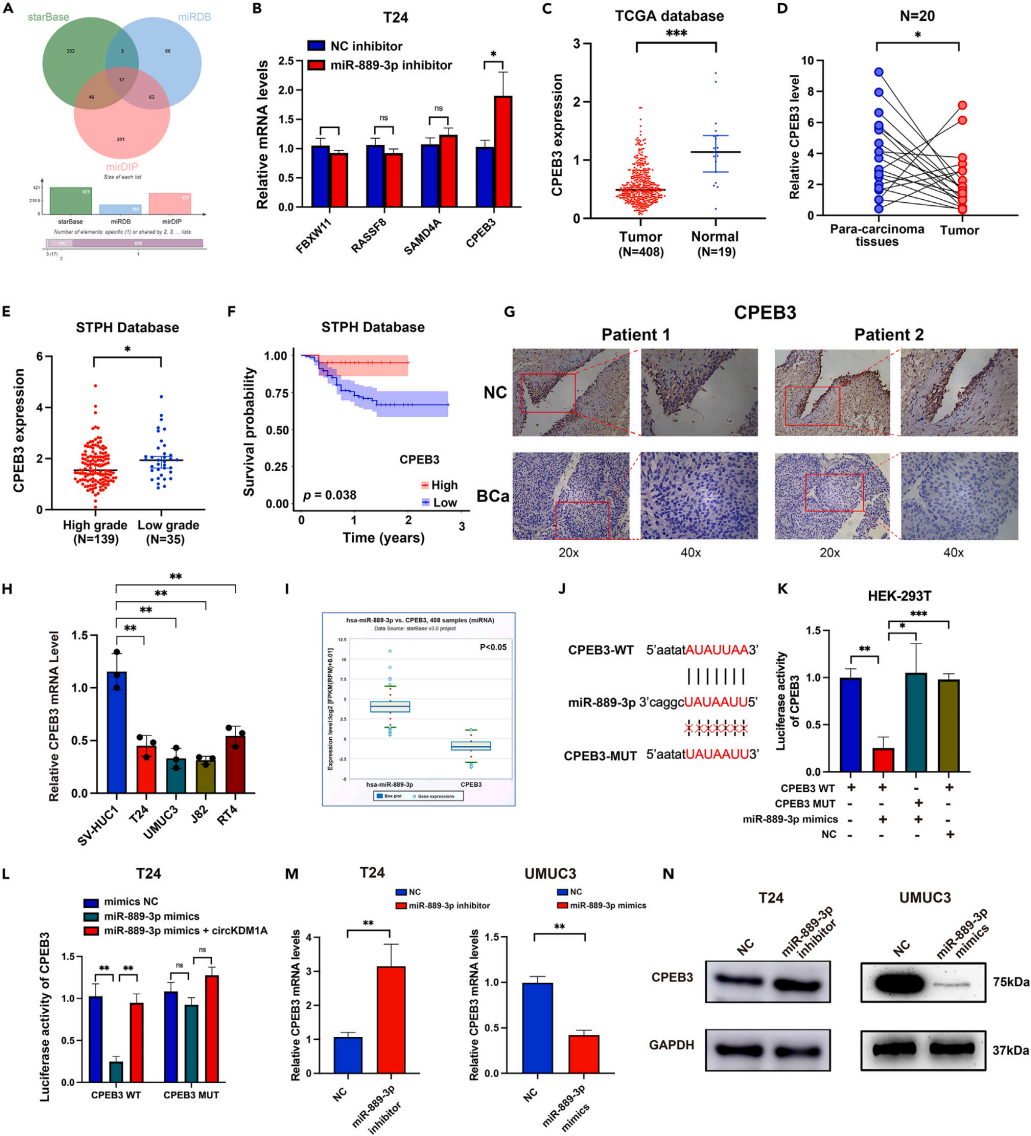
CPEB3 functions as the target of miR-889-3p in BCa (A) Venn diagram showing the mutual putative target genes of miR-889-3p. (B) qPCR confirmed that the expression of CPEB3 increased significantly after inhibiting miR-889-3p. (C) The expression of CPEB3 was low in BCa in the TCGA database. In the TCGA database, the expression of CPEB3 in BCa (n = 408) was significantly lower than in the normal group (n = 19). (D) qPCR demonstrated that CPEB3 levels were significantly decreased in BCa tissues from the STPH database (n = 20). (E) The expression of CPEB3 was significantly increased in low grade BCa (n = 35) compared to high grade (n = 139) in the STPH database. (F) Survival curve showed that high expression of CPEB3 (n = 20) was associated with better prognosis (Low expression CPEB3, n = 154). (G) The IHC staining of CPEB3 in patients of BCa and normal tissues. (H) qPCR showed that CPEB3 was low expressed in BCa cell lines. (I) The negative correlation between CPEB3 and miR-889-3p was predicted through the starBase (n = 408). (J) Schematic of CPEB3 wild-type (WT) and mutant (Mut) luciferase reporter vectors. (K) Dual luciferase reporter shows that CPEB3 can directly bind to miR-889-3p. (L) Overexpression of circKDM1A can restore the effect of miR-889-3p in inhibiting CPEB3 WT luciferase activity. (M) CPEB3 was negatively correlated with miR-889-3p verified by qPCR. (N) Western blot confirmed that overexpression or knockdown miR-889-3p can significantly change the expression of CPEB3.The data were presented as the mean ± SEM obtained from at least three independent experiments. Statistical significance was determined using either Student’s t test or one-way ANOVA as appropriate. ∗p < 0.05, ∗∗p < 0.01, ∗∗∗p < 0.001.

We searched Starbase, miRDB, and mirDIP databases to identify the possible target genes of miR-889-3p and found 17 candidate genes (Figure 5A). By further analyzing the expression and prognosis of 17 candidate genes, CPEB3, RASSF8, SAMD4A, and FBXW11 were identified as possible target genes of miRNA-889-3p. After transfecting T24 cells with the miR-889-3p inhibitor, only CPEB3 showed a significant increase (Figure 5B). Indeed, CPEB3 expression was low in the TCGA database and clinical tumor tissues (Figures 5C and 5D). We also assessed CPEB3 expression in low-grade BCa and observed this gene had high expression associated with better survival in the SHTP database (Figures 5E and 5F). Besides, IHC staining was used to demonstrate that CPEB3 expression was significantly higher in normal patient tissues than in BCa tissues (Figure 5G). qPCR results showed that CPEB3 expression was markedly reduced in the BCa cell lines compared with SV-HUC-1 cells (Figure 5H). These results suggest that CPEB3 may be a tumor suppressor gene of BCa. The Starbase database indicated a negative correlation between the expression of miR-889-3p and CPEB3 (Figure 5I; p < 0.05). To further verify that miR-889-3p can bind to CPEB3, HEK-293 T cells were performed to detect dual-luciferase activity. The results showed miR-653-5p mimic significantly reduced the luciferase activity of the CPEB3 WT (Figures 5J and 5K). Furthermore, overexpression of circKDM1A can restore the effect of miR-889-3p in inhibiting CPEB3 WT luciferase activity (Figure 5L; Figure S4A). Likewise, the expression of CPEB3 mRNA and protein increased in BCa cells treated with the miR-889-3p inhibitor by qPCR and Western blotting (Figures 5M and 5N; Figure S4B).

**Figure 6:**
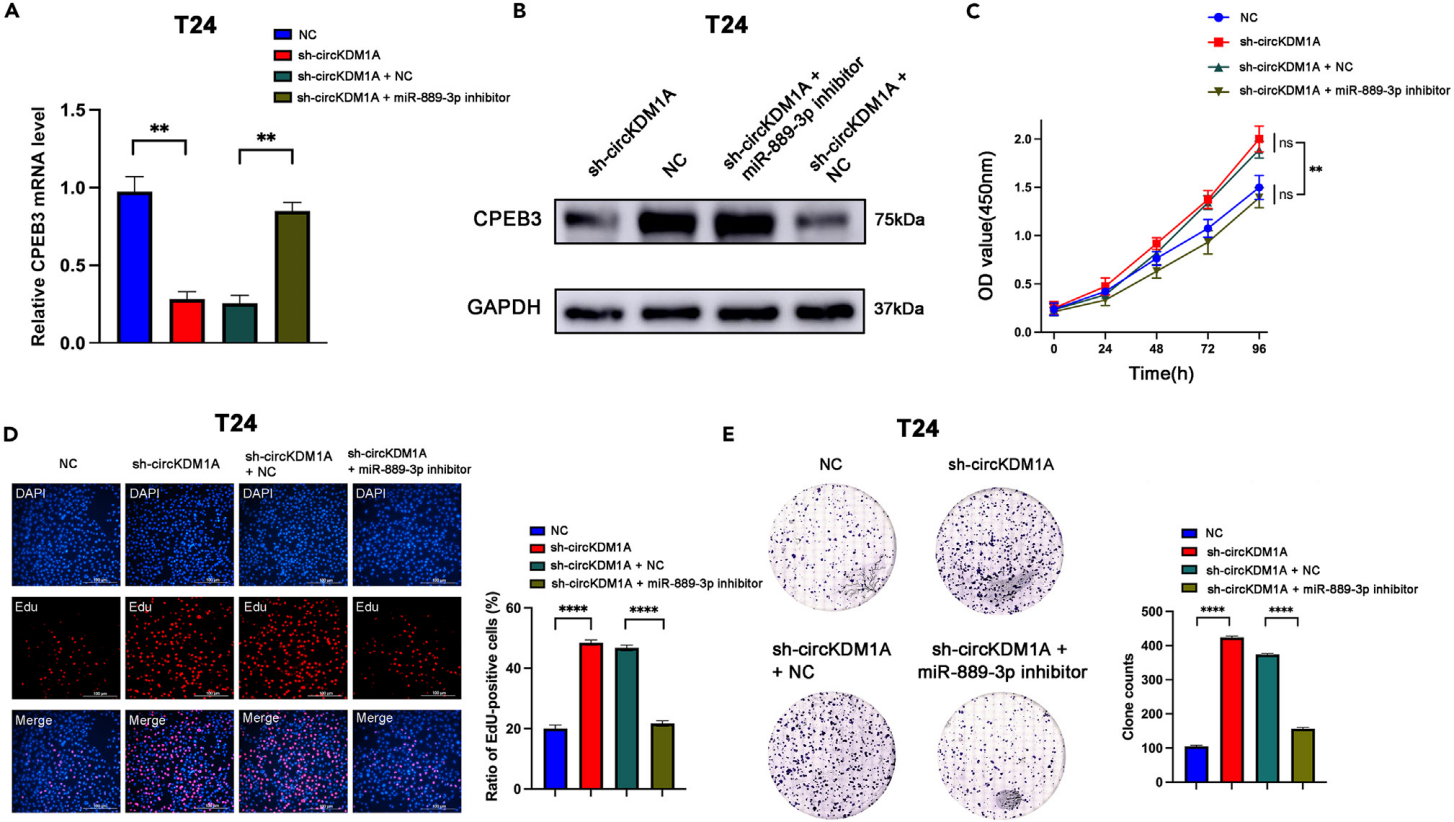
Inhibiting miR-889-3p can rescue BCa cell proliferation caused by knockdown circKDM1A (A) qPCR assays showed that miR-889-3p inhibitor reversed decreased CPEB3 mRNA levels in T24 cells. (B) Western blotting assays showed that the addition of miR-889-3p inhibitor to the circKDM1A knockdown group restored CPEB3 protein levels. (C) CCK-8 assay indicated that the inhibition of miR-889-3p could restore cell proliferation, which had been promoted by the knockdown circKDM1A. (D) EdU assay showed that miR-889-3p inhibitor can significantly reduce cell proliferation caused by knockdown circKDM1A. (E) Colony formation assay revealed that the inhibition of miR-889-3p can significantly decrease cell proliferation resulting from the knockdown of circKDM1A.The data were presented as the mean ± SEM obtained from at least three independent experiments. Statistical significance was determined using either Student’s t test or one-way ANOVA as appropriate. ∗p < 0.05, ∗∗p < 0.01, ∗∗∗p < 0.001, ∗∗∗∗p < 0.0001.

We performed rescue experiments by treating circKDM1A knockdown T24 cells with the miR-889-3p inhibitor to investigate whether circKDM1A inhibits BCa progression via CPEB3. The qPCR and Western blotting assays showed that miR-889-3p inhibitor reversed decreased CPEB3 mRNA and CPEB3 protein levels in T24 cells (Figures 6A and 6B; Figures S4C and S4D). The results of CCK-8, colony formation, and Edu assays confirmed this effect: the malignant proliferation of BCa cells was reversed by the miR-889-3p inhibitor (Figures 6C–6E; Figures S4E and S4F). In conclusion, our results demonstrate that circKDM1A downregulates CPEB3 by targeting miR-889-3p.

### Cytoplasmic polyadenylation element 3 affects the stability of p53 mRNA by binding to the cytoplasmic polyadenylation element signal of p53 3′ untranslated region

**Figure 7:**
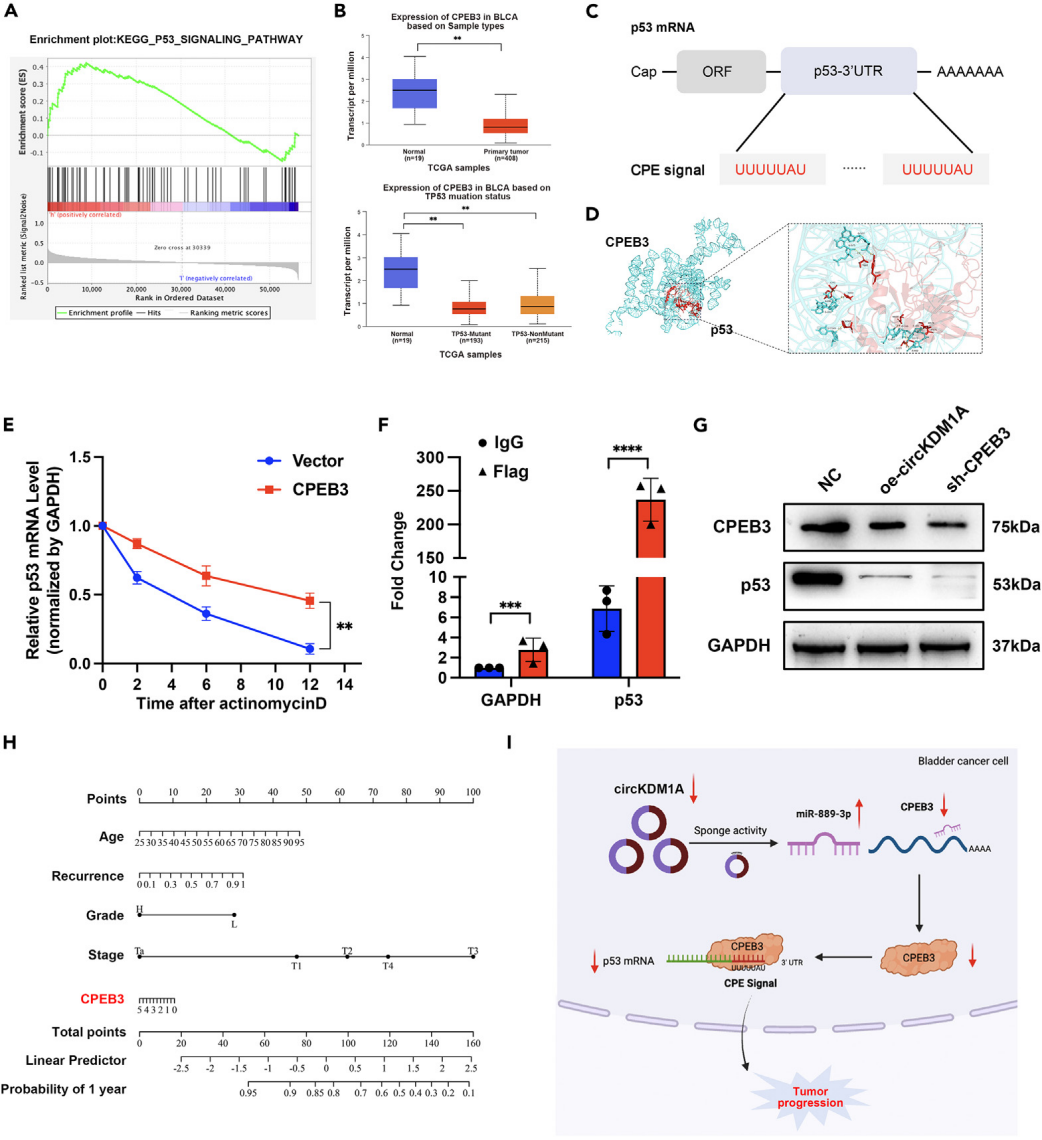
CPEB3 can affect the stability of p53 mRNA by binding to the CPE signal of p53 3′-UTR (A) GSEA enrichment analysis showed that the expression of CPEB3 was significantly positively correlated with the p53 pathway. (B) CPEB3 expression was lower in patients with p53 mutations (n = 193). (C) The p53 mRNA 3′ UTR contains two CPE signals (UUUUAU), suggesting that CPEB3 may regulate the activation or repression of p53 mRNA. (D) 3D structural diagram of CPEB3 protein binding to p53 mRNA 3′UTR by PyMOL software. (E) Actinomycin D experiments showed that the overexpression of CPEB3 could significantly inhibit the degradation of p53 mRNA. (F) RIP-qPCR experiments showed that the CPEB3 protein can directly bind p53 mRNA. (G) Western blot results showed that the overexpression of circKDM1A or knocking down CPEB3 could reduce the expression of p53. (H) A nomogram constructed based on the expression of CPEB3 and the clinicopathological data of patients in the STPH database. (I) The proposed model of the mechanism by which circKDM1A can inhibit the expression of CPEB3 through the molecular sponge mechanism, thereby inhibiting the expression of p53 and promoting tumor progression.The data were presented as the mean ± SEM obtained from at least three independent experiments. Statistical significance was determined using either Student’s t test or one-way ANOVA as appropriate. ∗p < 0.05, ∗∗p < 0.01, ∗∗∗p < 0.001.

We conducted a GSEA analysis to investigate the molecular mechanism of CPEB3 affecting BCa progression, finding that CPEB3 expression was closely related to the p53 pathway (Figure 7A). After analyzing the TCGA database, we observed that CPEB3 expression was significantly lower in the p53-mutant group than in normal tissues. This intriguing result suggests that low CPEB3 expression downregulates the tumor suppressor p53 gene in BCa (Figure 7B). At the mechanistic level, we found that the 3′ UTR of p53 contains the cytoplasmic polyadenylation element (CPE) with the UUUUAU sequence, indicating that p53 is a target of CPEB3 (Figure 7C). We confirmed this hypothesis by visualizing the 3D structure of CPEB3 protein binding p53 mRNA 3′ UTR CPE signal using PyMOL software (Figure 7D; Figure S5A). Furthermore, we treated CPEB3 overexpression cell lines with actinomycin D to assess p53 mRNA stability. The result showed that CPEB3 overexpression inhibited the actinomycin D-induced degradation of p53 mRNA (Figure 7E). This result suggests that CPEB3 affects the stability of p53 mRNA. Finally, we used a CPEB3 antibody to pull down p53 mRNA and validated its expression with qPCR, confirming that CPEB3 directly binds p53 mRNA. (Figure 7F).

Western blotting showed that knocking down CPEB3 protein lowered p53 expression (Figure 7G), reinforcing that CPEB3 upregulates p53 mRNA expression by binding to its 3′ UTR and increasing the stability and translation of p53 mRNA. Therefore, combined with the clinical sequencing data of our center and the clinical information of patients, we also constructed a nomogram combining the sequencing data from our clinical center and the clinical information of patients (Figure 7H). The nomogram estimated the value of CPEB3 expression in predicting the accurate diagnosis of patients with BCa (Figures S5B–S5D).

## Discussion

In this study, we discovered a new circRNA tumor suppressor called circKDM1A and showed it was downregulated in BCa cell lines and tissues. Low circKDM1A expression is significantly associated with poor prognosis of BCa. Mechanistically, circKDM1A acts as a molecular sponge binding miR-889-3p and achieving a regulatory effect through CPEB3. Moreover, the CPEB3 protein directly binds to the CPE signal of p53 mRNA 3′ UTR to affect the p53 mRNA stability (Figure 7I).

The malignant proliferation of tumor cells is the main feature of almost all cancers, and patients with tumors with higher proliferation capacity usually have worse prognosis.^18^^,^^19^ The BCa chemotherapy drugs that are currently in clinical use, such as gemcitabine and epirubicin, exert anti-cancer effects by inhibiting the proliferation of tumor cells.^20^^,^^21^ There is considerable evidence that circRNAs play a role in regulating the malignant proliferation of tumor cells.^22^ For example, circ_0000140 inhibited the growth of oral squamous cell carcinoma cells through the miR-31/LATS2 axis,^23^ while circMTO1 impeded the proliferation of liver cancer cells through the miR-9/p21 axis.^24^ At present, there is limited knowledge regarding the function of circKDM1A in tumors. Song et al. reported that the expression of circKDM1A was significantly down-regulated in prostate cancer tissues and was associated to low stages.^25^ Although some research has been conducted on circKDM1A, the underlying mechanism by which it regulates tumor progression has not been thoroughly investigated. In this study, we performed *in vivo* and *in vitro* experiments to demonstrated that differentially expressed circKDM1A in BCa significantly promotes cancer cell proliferation. We also discovered that the circKDM1A effect on cell proliferation relies on a mechanism that regulates the classic tumor suppressor gene p53.

CircRNAs contain a high concentration of miRNA response elements and can competitively bind to miRNAs to form a ceRNA regulatory network.^26^ MiRNAs are a type of non-coding RNA that are approximately 22 nt in length. They play a crucial role in post-transcriptional regulation by binding to specific sites in the 3′ untranslated region of mRNA.^27^ ln addition to competitively binding circKDM1A, miR-889-3p also binds several other circRNAs. For instance, in cervical cancer, hsa_circ_0009035 promotes tumor progression and radioresistance via the miR-889-3p/HOXB7 axis.^28^ In gastric cancer, circDYRK1A restrains glutamine metabolism by up-regulating miR-889-3p-dependent F box protein 4 (FBXO4).^29^ Therefore, mir-889-3p seems to hold a vital role in the circRNA sponging mechanism.

The CPEB protein family encompasses 4 homologous proteins.^30^ Its members recruit translation inhibitors to the CPE sequence element in the 3′ UTR region of target mRNAs, which, in turn, bind to the CPE canonical sequence (UUUUUAU) and regulate mRNA expression. The N-terminal structure of CPEBs is highly variable, while the C-terminal is relatively conserved.^31^ These proteins are widely expressed in tissues and tumors in partially overlapping patterns.^32^ Our study is the first to report and elucidate the function and mechanism of CPEB3 in BCa. The protein has significantly low expression in BCa and is associated with a poor prognosis in patients with the disease. Moreover, our study also revealed that circKDM1A/miR-889-3p regulates the CPEB3 expression through a circRNA-miRNA molecular sponging mechanism.

Previous research has primarily concentrated on the effect of p53 mutations and ubiquitination on BCa.^33^^,^^34^ Shi et al. discovered that in a p53 mutant background, YY1 stimulates PHGDH expression and promotes bladder tumorigenesis.^35^ The lncRNA LNPPS obstructs PDCD5 ubiquitination and competitively inhibits MDM2-mediated p53 ubiquitination, thereby promoting PDCD5/p53-related cell apoptosis.^36^ Our study is the first to describe the function of CPEB3 and the mechanism by which circKDM1A regulates p53 in BCa. Specifically, the 3′ UTR of p53 mRNA contains highly conserved CPE sites located immediately upstream of the polyadenylation sequence, consistent with the D'Erchia et al. study.^37^ Through actinomycin D application experiments, we preliminarily showed that CPEB3 may regulate the stability and translation of p53 mRNA by binding to the CPE site in the 3′ UTR of p53 mRNA.

In this study, circKDM1A was significantly low-expressed in BCa and could regulate p53. At the same time, circRNAs are more stable than linear RNA, so we believe they will have clinical application and translational value in the future. Our group has developed an electrochemical biosensor that can directly detect circRNA in micro-volume urine samples (25 μL) for the diagnosis of BCa.^38^ Next, we plan to combine circKDM1A and the biosensor to further improve the efficiency of BCa diagnosis.

In conclusion, our study is the first to show that circKDM1A is downregulated in BCa and acts as a cancer suppressor. Low circKDM1A expression upregulates miR-889-3p, inhibiting the CPEB3/p53 axis and promoting BCa malignant proliferation. Thus, our findings provide new insights into the molecular mechanisms of BCa cell proliferation and potential biomarkers and therapeutic targets for BCa.

### Limitations of the study

This study has several limitations that need to be addressed in future research. First, larger samples and multi-center studies are needed to clarify the impact of circKDM1A on the poor prognosis of BCa. Second, we did not construct a CPE mutation of p53 mRNA 3′-UTR, which requires further experimental verification. Third, the underlying mechanism of low expression of circKDM1A still needs further exploration.

## STAR★Methods

### Key resources table

**Table:** 

REAGENT or RESOURCE	SOURCE	IDENTIFIER
**Antibodies**		

Bax	Abcam	Cat# ab32503; RRID:AB_725631
Bcl2	Abcam	Cat# ab182858; RRID:AB_2715467
Cyclin D1	Abcam	Cat# ab16663; RRID:AB_443423
GAPDH	Abcam	Cat# ab181602; RRID:AB_2630358
CPEB3	Abcam/proteintech	Cat# ab10883/KHC1835; RRID:AB_10895013
p21	Abcam	Cat# ab109520; RRID:AB_10860537
p53	Abcam	Cat# ab26; RRID:AB_303198
Flag	Abclonal	Cat# AE005; RRID:AB_2770401

**Biological samples**		

Human BCa tissues, adjacent normal tissues	Shanghai Tenth People’s Hospital of Tongji university	N/A

**Chemicals, peptides, and recombinant proteins**		

RPMI Medium 1640	Yeasen	Cat# 41402ES76
DMEM	Yeasen	Cat# 41420ES76
F12-K	Bosterbio	Cat# PYG0093
Fetal bovine serum	Gibco	Cat# 10099158
DMSO	Yeasen	Cat# 0203ES76
TRIzol	Invitrogen	Cat# 15596018CN
Actinomycin D	Yeasen	Cat# 40722ES03
RNase R	Yeasen	Cat# 14606ES72

**Critical commercial assays**		

BCA Assay Kit	Yeasen	Cat# 20201ES76
qPCR SYBR Green Master Mix kit	Yeasen	Cat# 11200ES03
HiScript III 1st Strand cDNA Synthesis Kit	Vazyme	Cat# R312-01
PARI kit	ThermoFisher	Cat# AM1921
RNase R enzyme	Yeasen	Cat# 14606ES72
Lipofectamine 3000 Kit	Invitrogen	Cat# L3000001
Dual luciferase reporter assay system Kit	Yeasen	Cat# 11402ES60
DAB kit	Beyotime	Cat# P0203
RIP kit	BersinBio	Cat# Bes5101 (N)
EdU imaging kit	APExBIO	Cat# K1075
CCK-8 Kit	Yeasen	Cat# 40203ES60

**Deposited data**		

Microarray dataset	N/A	GEO: GSE92675
Starbase	N/A	http://starbase.sysu.edu.cn/
CircInteractome	N/A	https://circinteractome.irp.nia.nih.gov/
CircBank	N/A	https://www.circbank.cn/
miRDB	N/A	https://mirdb.org/
mirDIP	N/A	http://ophid.utoronto.ca/mirDIP/index.jsp

**Experimental models: Cell lines**		

T24	ATCC	Cat# HTB-4
UMUC3	ATCC	Cat# CRL-1749
J82	ATCC	Cat# HTB-1
RT-4	ATCC	Cat# HTB-2
HEK293T	ATCC	Cat# CRL-3216
SV-HUC-1	ATCC	Cat# CRL-9520

**Experimental models: Organisms/strains**		

Female BALB/c nude mice	SLAC	N/A

**Oligonucleotides**		

Primer sequences	See Table S1	N/A
RNA pull-down probe	See Table S2	N/A

**Software and algorithms**		

GraphPad Prism 9.0	GraphPad	N/A
SPSS 26.0	IBM SPSS	N/A
ImageJ	NIH ImageJ	N/A

### Resource availability

#### Lead contact

Further information and request for resources and reagents should be directed to and will be fulfilled by the lead contact, Xudong Yao (yaoxudong1967@163.com/yxd@tongji.edu.cn).

#### Materials availability

This study did not generate new unique reagents.

#### Data and code availability

The data in the present study can be obtained from the public datasets specified in the key resources table. All data reported in this paper will be shared by the lead contact upon request.

This paper does not report original code.

Any additional information required to reanalyze the data reported in this paper is available from the lead contact upon request.

### Experimental model and study participant details

#### Ethics approval and consent to participate

The Ethics Review Committee of Shanghai Tenth People’s Hospital (STPH) granted approval for this research (approval No. 20190203). The animal experiments were conducted in compliance with the regulations set by the Experimental Animal Ethics Committee of STPH (SHDSYY-2023-3028).

#### Human subject

Patients with BCa from the STPH, Tongji University, China, provided 20 pairs of BCa tissues and their matched para-carcinoma tissues (Table S3). The tissue samples were preserved immediately in liquid nitrogen after collection. The Institutional Ethics Committees of STPH approved the study (approval No. 20190203), and all patients or their relatives provided informed consent before the sample collection. A dataset from STPH was obtained from our clinical database with clinicopathological and transcriptome information on 174 patients with BCa enrolled from November 2019 to July 2022 and used for bioinformatic analyses (Table S4). The protocols for total RNA extraction, paired-end library generation, and RNA sequencing performed to obtain the STPH dataset were similar to those published in our earlier study.^39^ Disease-free survival (DFS) in patients with BCa was expressed as the time from surgical intervention to first recurrence or first progression (including metastasis or death).

#### Animals

Four-week-old female Bale/c nude mice were acquired from SLAC in Shanghai, China and kept in a pathogen-free environment. The animal experiments were conducted in compliance with the regulations set by the Experimental Animal Ethics Committee of STPH (SHDSYY-2023-3028).

#### Cell lines

The Shanghai Institutes for Biological Sciences, Chinese Academy of Sciences, provided the cell lines for this study: human normal bladder epithelial cell line, SV-HUC-1; BCa cell lines, T24, UMUC3, J82, RT-4; and human embryonic kidney cell HEK293T. The cells were cultured in the following media: SV-HUC-1, F12-K medium; RT-4, McCoy’s 5A medium; HEK-293T, DMEM medium; T24, UMUC3, and J82, RPMI 1640 medium. All cell lines were cultured with 10% fetal bovine serum (FBS) (Gibco, USA) and 1% penicillin-streptomycin at 37°C with 5% CO_2_.

### Method details

#### Quantitative PCR (qPCR)

Total RNA was extracted from frozen tissues and cultured cells with Trizol reagent (Invitrogen, USA). It was reverse transcribed into cDNA using the HiScript III 1st Strand cDNA Synthesis Kit (Vazyme, China) following the manufacturer’s instructions. The qPCR assay was performed using the Hieff UNICON qPCR SYBR Green Master Mix kit (Yeasen, China), and primer sequences were designed and synthesized by Sangon Biotech (Shanghai, China) (Table S1).

#### Fractionation of nuclear and cytoplasmic RNA

Nuclear and cytoplasmic RNAs were extracted using a PARI kit (ThermoFisher, USA) according to the manufacturer’s protocol. The relative RNA abundance in different cell fractions was detected by qPCR. The relative expression of nuclear RNAs was normalized to that of U6 endogenous control, while the expression of cytoplasmic RNAs was compared with that of GAPDH.

#### RNase R linear RNA digestion

Total RNA was isolated, and 20 μg of the freshly extracted RNA was divided into 2 equal aliquots. The first was treated with RNase R enzyme (Yeasen, China) as the experimental group, whereas the second was mixed with the same amount of 10 × RNase R reaction buffer as the control group. After digestion at 37°C for 30 min, the digested RNA was reverse transcribed with specific primers and quantified by qPCR.

#### Cell transfection

Lipofectamine 3000 (Invitrogen, USA) was applied following the manufacturer’s instructions to transfect T24 and UMUC3 cells. Plasmid and lentivirus expression vector constructs for sh-circKDM1A or oe-circKDM1A stable cell lines, miR-889-3p mimic, and miR-889-3p inhibitor were designed and synthesized by Invitrogen.

#### Cell counting kit-8 (CCK-8) and colony formation assays

A single-cell suspension containing 2000 cells/ml was prepared, and cells were seeded onto 96-well plates at 200 μL cells/well. During incubation, cells were quickly treated with 10 μL of CCK-8 solution and 100 μL of RPMI 1640 stock solution at 24 h, 48 h, 72 h, or 96 h. They were placed back to the incubator at 37°C incubator for an appropriate time, and the absorbance of each well was read at 450 nm.

Cells were plated onto 6-well plates at a density of 1000 cells/well. The formed cell colonies were washed 2× with cold phosphate-buffered saline, fixed with 95% ethanol, and stained with 0.1% crystalline purple for 15 min. The colonies were counted and photographed.

#### 5-Ethynyl-2′-deoxyuridine (EdU) and fluorescence *in situ* hybridization (FISH) assays

For EdU staining, 50,000 cells were seeded onto the cell creeper. The next day, the cell creeper was washed 3× with precooled PBS and stained according to the instructions described inside an EdU imaging kit (Cy3). The nuclei were stained with Hoechst reagent. The experimental results were recorded with a fluorescence microscope.

The FISH experiments were also performed on cell slides. The circKDM1A and the 18S probes were designed by Sangon Biotech (Shanghai, China). The subcellular location of circKDM1A was determined by staining BCa cells following the FISH kit protocol. The 18S probe was as a positive control for the nuclei.

#### Western blotting

Cells were lysed using a radioimmunoprecipitation assay buffer (Beyotime, Shanghai, China). The concentrations of the proteins in the lysates were assessed with the bicinchoninic acid assay. Proteins were separated by applying the lysates onto 10% sodium dodecyl sulfate-polyacrylamide gels and transferring them onto nitrocellulose membranes (Yeasen, China). The membranes were blocked with 5% non-fat milk for 1 h and probed with primary antibodies at 4°C overnight. A 1-h incubation with secondary antibodies followed at room temperature. The membranes were exposed using a Tanon 5200 ECL imaging system (Tanon, Shanghai, China). The information on the antibodies is listed in the key resources table.

#### Subcutaneous xenograft tumor model

A subcutaneous xenograft tumor model was established using 10 female nude mice aged 4 weeks. The animals were assigned into 2 groups (n = 5), and 5 × 10^6^ T24 cells expressing short hairpin (sh)-circKDM1A or negative control (NC) were injected subcutaneously into the right armpit of the upper limb of each animal. Tumor size was measured and calculated every 4 days. After 4 weeks, the mice were euthanized, and the tumors were collected.

#### Dual-luciferase reporter assay

Wild-type or mutant reporter plasmids carrying circKDM1A and CPEB3 were designed and synthesized by Yeasen (China) to confirm that miR-889-3p directly targets circKDM1A and CPEB3. The plasmids were introduced into HEK293T cells by co-transection with miR-889-3p mimics or miR-889-3p-NC. Luciferase activities were measured using a microplate reader, followed by calculating firefly to Renilla luciferase ratios.

#### RNA immunoprecipitation (RIP)

The circKDM1A and circKDM1A-binding miRNA pulldowns were performed using an RIP kit (BersinBio, Guangzhou, China). The biotinylated circKDM1A probe for pulling down miRNAs were synthesized by Shanghai GenePharma (Shanghai, China). In brief, T24 cells were harvested and lysed with lysis buffer. The probes were incubated with streptavidin-coated magnetic beads to generate probe-coupled magnetic beads. The cell extract was incubated with magnetic beads coupled with the circKDM1A or NC probe to pull down miRNAs. Pulled-down miRNAs were collected using an RNA elution buffer and evaluated with qPCR for identification. Potentially bound RNA was pulled down following the RIP kit protocol, captured with an IP-grade CPEB3 antibody, and validated by qPCR. IgG served as a negative control. The information of probes was described in Table S2.

#### Predicting 3D structures for molecular docking

Download the protein three-dimensional structure file CPEB3_HUMAN (UniProt ID: Q8NE35) predicted by AlphaFold from UniProt to predict the RNA three-dimensional structure.^40^ Then perform protein-RNA molecular docking^41^ or RNA-RNA molecular docking.^42^ Select the best binding model, use PyMOL v2.5.4 to analyze polar interactions, and draw a 3D interaction diagram.^40^ The yellow dotted line represents the hydrogen bond interaction that occurs between protein amino acid residues and RNA bases.

#### Immunohistochemistry (IHC)

The sections from BCa tissues or normal tissues were dehydrated and were treated with appropriate dilution of the CPEB3 primary antibody (1:50) at 4°C overnight. Next, 100μL HRP-labelled second antibodies at a dilution of 1:500 was added for 60 min of incubation at 37°C. The positive staining was visualized by a DAB kit (P0202, Beyotime, Shanghai, China) and hematoxylin (C0107, Beyotime, Shanghai, China). The staining results were photographed with a microscopy (Nikon, Japan).

#### Bioinformatic analysis

A microarray dataset GSE92675 was retrieved from the Gene Expression Omnibus database (https://www.ncbi.nlm.nih.gov/geo/) using the GPL19978 platform and included 4 BCa and paired adjacent tissue samples. Differentially expressed circRNAs in BCa were identified using the “limma” package and visualized using the "ggplot2" and "pheatmap" packages. All the packages were used in R (version 4.2.2). The circRNA-binding miRNAs were predicted using the web tools CircInteractome,^43^ Starbase,^44^ and CircBank.^45^ The miR-889-3p-binding target genes were predicted using the miRDB,^46^ mirDIP,^47^ and StarBase databases.

### Quantification and statistical analysis

#### Statistical analysis

The statistical significances between the groups were assessed by GraphPad Prism version 9.0 (GraphPad, CA, USA) and SPSS version 26.0 (IBM, Armonk, NY, USA). Comparisons between 2 groups were analyzed with the Student’s t test and one-way analysis of variance (ANOVA) was used for comparisons across multiple groups. Correlation analyses were performed using Spearman correlation test. The mean ± SEM was used to express the experimental data. Statistical significance was inferred when p < 0.05. ∗p < 0.05, ∗∗p < 0.01, ∗∗∗p < 0.001, ∗∗∗∗p < 0.0001.

## Supplemental information

Document S1. Figures S1–S5 and Tables S1–S4

Data S1. WB original data
