# Supplementary material for: circKDM1A suppresses bladder cancer progression by sponging miR-889-3p/CPEB3 and stabilizing p53 mRNA
Source: iScience. 2024 Mar 29;27(4):109624. doi: 10.1016/j.isci.2024.109624 (PMC11022052; doi:10.1016/j.isci.2024.109624)
Supplement: Document S1. Figures S1–S5 and Tables S1–S4 [file mmc1.pdf]

iScience, Volume *27*

Supplemental information

circKDM1A suppresses bladder cancer progression by sponging miR-889-3p/CPEB3 and stabilizing p53 mRNA

Haotian Chen, Jing Wen, Wentao Zhang, Wenchao Ma, Yadong Guo, Liliang Shen, Zhijin Zhang, Fuhan Yang, Yue Zhang, Yaohui Gao, Tianyuan Xu, Yang Yan, Wei Li, Junfeng Zhang, Shiyu Mao, and Xudong Yao

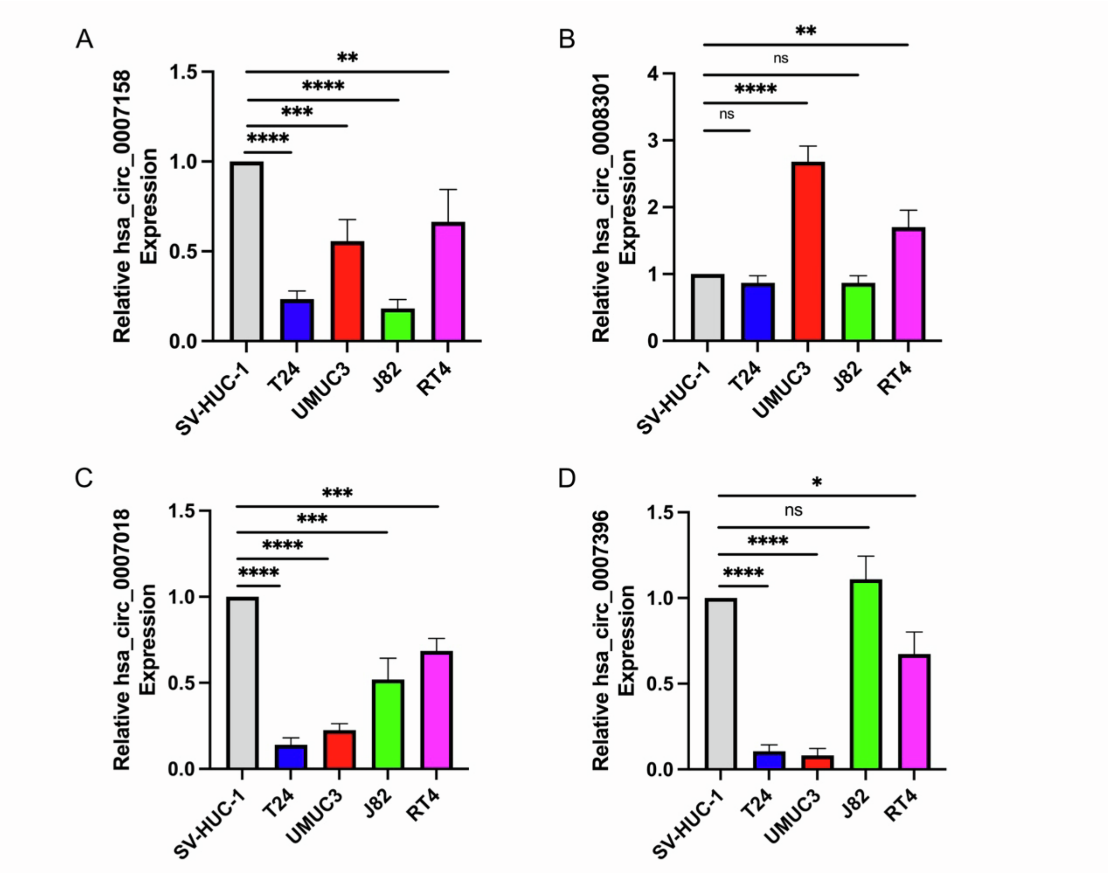

Figure S1. The expression abundance of 4 screened circRNA candidates. Related to Figure 1.

(A-D) qPCR showed the expression of (A) hsa_circ_0007158, (B) hsa_circ_0008301, (C) hsa_circ_0007018, (D) hsa_circ_0007396.

Data are represented as mean ± SD. *p < 0.05, **p < 0.01, ***p < 0.001, **** p < 0.0001.

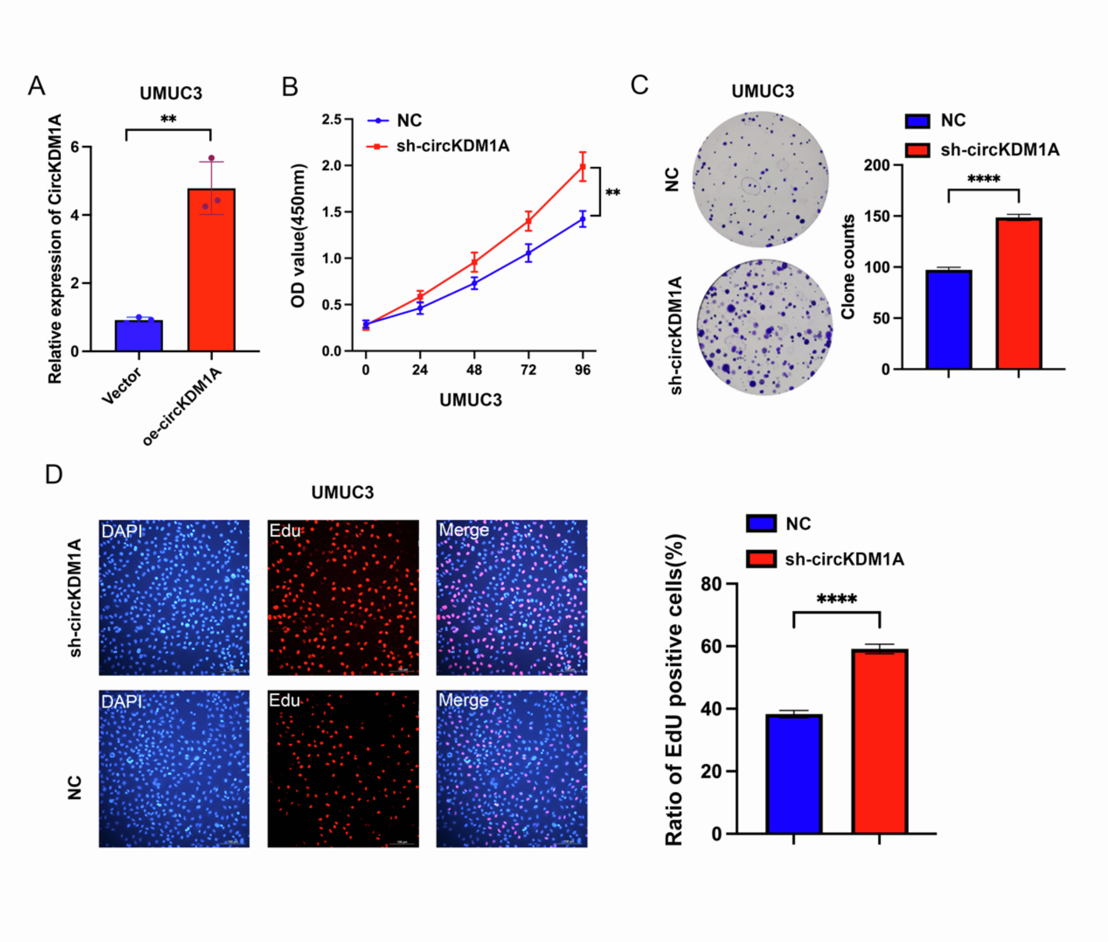

Figure S2. Sh-circKDM1A promotes proliferation of bladder cancer cell line UMUC3. Related to Figure 3.

(A) The knockdown efficiency of circKDM1A was verified by qPCR.

(B-D) CCK-8 assay, clone formation assay, and EdU assay showed that knockdown of circKDM1A promoted bladder cacner cell proliferation.

Data are represented as mean ± SD. *$p < 0.05$, **$p < 0.01$, ***$p < 0.001$, **** $p < 0.0001$.

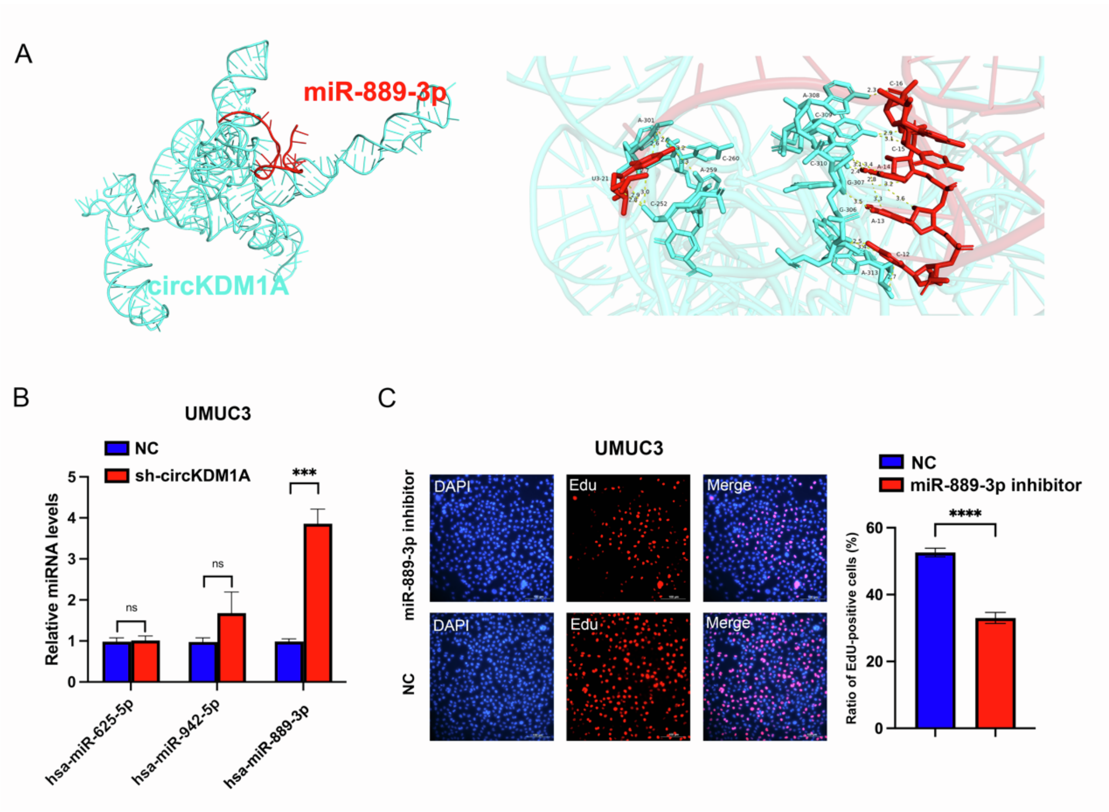

Figure S3. Predict the binding site of circKDM1A and miR-889-3p and verify the knock down efficiency of circKDM1A in UMUC3. Related to Figure 4.

(A) PyMOL software displays the interaction between circKDM1A and miR-889-3p and base pair structure.

(B) qPCR showed that the expression of miR-889-3p was significantly increased after knocking down circKDM1A in UMUC3 cell line.

(C) EdU experiments showed that miR-889-3p inhibitor suppressed the proliferation of UMUC3 BCa cells.

Data are represented as mean ± SD. $^*p < 0.05$, $^{**}p < 0.01$, $^{***}p < 0.001$, $^{****}p < 0.0001$.

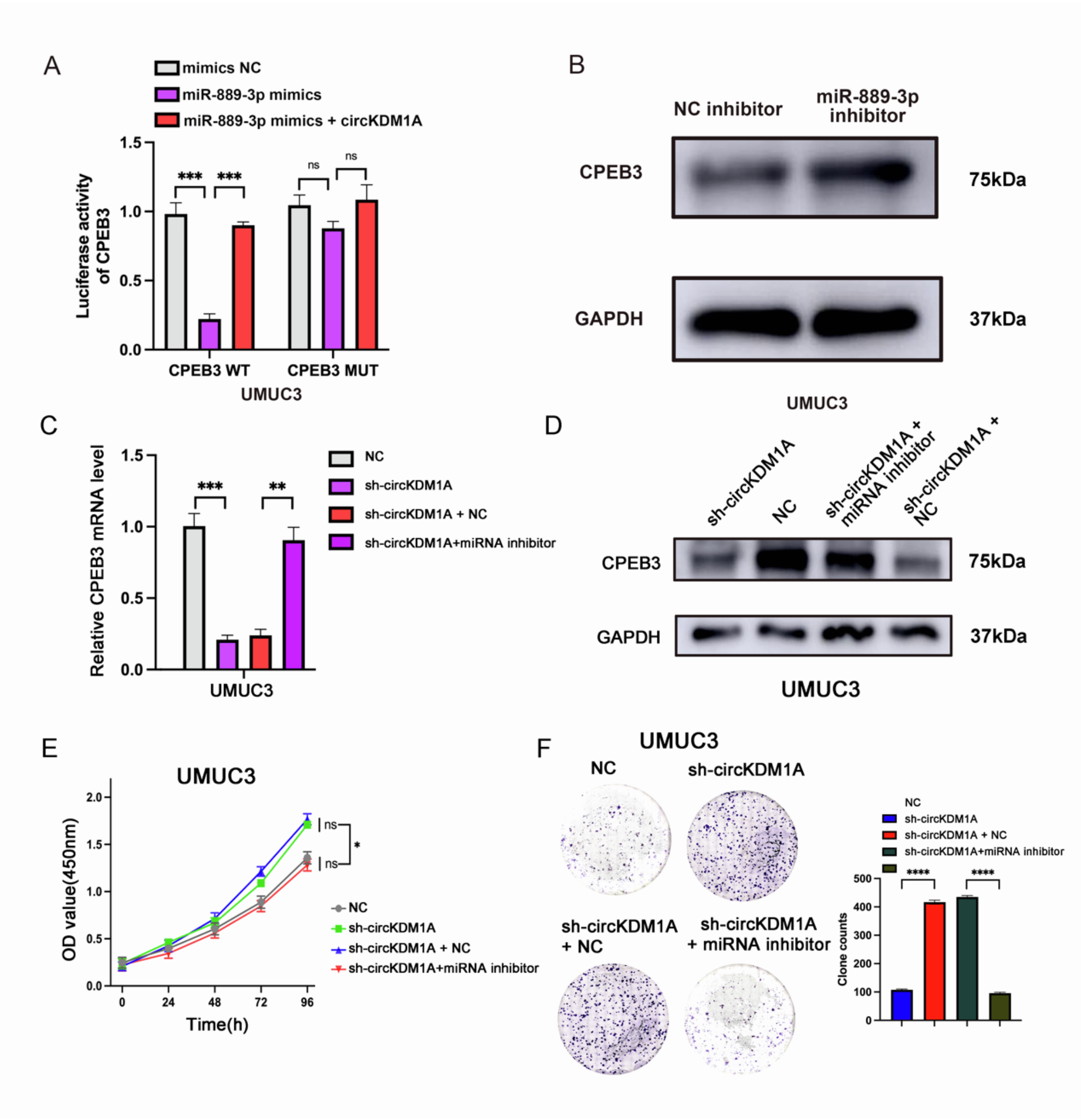

Figure S4. Verification of circKDM1A binding to miR-889-3p and rescue experiments. Related to Figure 5 and Figure 6.

(A) Dual luciferase reporter shows that CPEB3 can directly bind to miR-889-3p in UMUC3 cell line.

(B) Western blot confirmed that knockdown miR-889-3p can significantly change the expression of CPEB3.

(C-D) Rescue experiment showed that knockdown circKDM1A + miR-889-3p can evaluate the expression of CPEB3 in UMUC3 cell line.

(E) The CCK-8 assay indicated that the inhibition of miR-889-3p could restore cell proliferation, which had been promoted by the knockdown of circKDM1A in UMUC3.

(F) Colony formation assay showed that miR-889-3p can significantly reduce cell proliferation caused by knockdown of circKDM1A in UMUC3.

Data are represented as mean ± SD. $*p < 0.05$, $**p < 0.01$, $***p < 0.001$, $****\ p < 0.0001$.

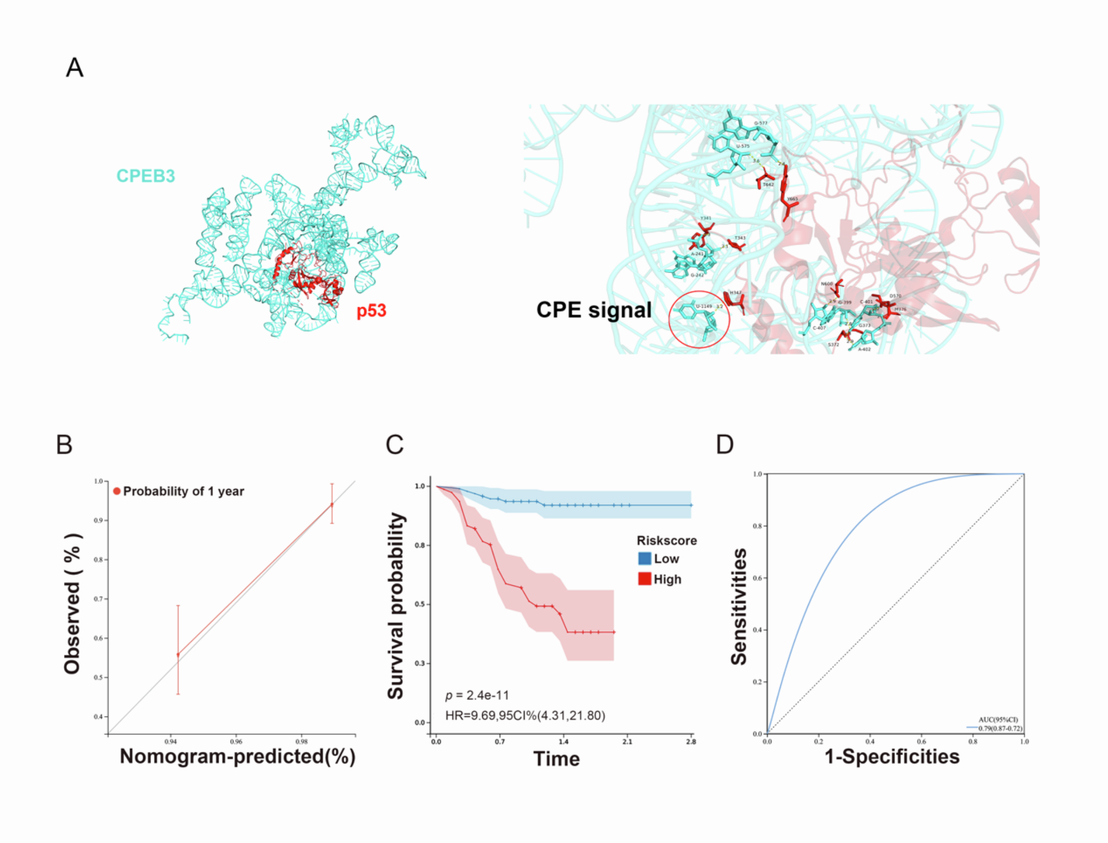

Figure S5. Predict the binding site of CPEB3 to p53 and establish a clinical prediction model based on CPEB3. Related to Figure 7.

(A) The binding of CPEB3 and p53 and the base position of CPE signal were predicted by PyMOL software.

(B) The calibration curve shows the high reliability of our Nomogram.

(C) The survival curve showed that patients with low CPEB3 expression had a worse prognosis.

(D) The ROC curve shows that the nomogram established based on CPEB3 has good predictive ability.

Table S1. Primer name and sequence involved in this manuscript. Related to Figure 1, 4, 5 and 7.

Primer name	Primer sequence (5'→3')
hsa_circ_0009061-F	CTCAAGAAGCAGCCTGTTTTC
hsa_circ_0009061-R	TTCATCCATCTCTCTGTACTCTACTGT
hsa_circ_0007158-F	TGCTGGAGGACTTTGTTGATT
hsa_circ_0007158-R	CAGGGAATGCCATCCTCTG
hsa_circ_0008301-F	CAGCCCGTGGTCCTGATG
hsa_circ_0008301-R	AGCTCACCGATGTACACCTGT
hsa_circ_0007018-F	TACTCTACAATCCATTTCCTCCC
hsa_circ_0007018-R	TACAGATAATGTTCTCAGGACCCTT
hsa_circ_0007396-F	TCTTCAAGCTGGAGCAGGTG
hsa_circ_0007396-R	TGAGGCTGTAGGGAAGGATAAG
B-actin Forward	CCTGGCACCCAGCACAAT
B-actin Reverse	GGGCCGGACTCGTCATAC
U6-RT	GTCGTATCGACTGCAGGGTCCGAGGTATTCGCAGTCGATACGA CAAAATATG
U6-Forward	AGCACATATACTAAAATTGGAACGAT
hsa-miR-942-5p RT	GTCGTATCCAGTGCAGGGTCCGAGGTATTCGCACTGGATACGA CCACATG
hsa-miR-942-5p Forward	CGCGTCTTCTCTGTTTTGGC
hsa-miR-889-3p RT	GTCGTATCCAGTGCAGGGTCCGAGGTATTCGCACTGGATACGA CACAATG
hsa-miR-889-3p Forward	GCGCGTTAATATCGGACAAC
hsa-miR-625-5p RT	GTCGTATCGACTGCAGGGTCCGAGGTATTCGCAGTCGATACGA CGGACTA
hsa-miR-625-5p Forward	CGGCAGGGGGAAAGTTCTA

Common R	ACTGCAGGGTCCGAGGTATT
KDM1A Forward	GACTTCTTGGCAGAGTTGTC
KDM1A Reverse	GTGAAAGAGTTGCAGATCC
CPEB3 Forward	TCAACACAACGACATTGACAAA
CPEB3 Forward	CCCTGACACTCGTCACACAT
p53 Forward	GGGAGCACTAAGCGAGCACTG
p53 Reverse	TTGGACTTCAGGTGGCTGGA

Table S2. The RNA pull-down probe sequence involved in this manuscript. Related to Figure 4.

RNA pull-down probe	Sense（5‘-3’）	
hsa_circ_0009061	GTACTCTACTGTGCGGTTTCTAATG	Biotin
Negative Control	TAGGGTCCTCTCGTTTATAGACTGT	Biotin

Table S3. The clinical information of 20 pairs of BCa tissues and their matched para-carcinoma tissues. Related to Figure 4 and 5.

Number	Age	Gender	Race	Ethnicity
1	68	Male	Asian	Han Chinese
2	70	Male	Asian	Han Chinese
3	61	Male	Asian	Han Chinese
4	75	Male	Asian	Han Chinese
5	73	Male	Asian	Han Chinese
6	71	Male	Asian	Han Chinese
7	64	Male	Asian	Han Chinese
8	62	Male	Asian	Han Chinese
9	67	Male	Asian	Han Chinese
10	82	Male	Asian	Han Chinese
11	75	Male	Asian	Han Chinese
12	77	Male	Asian	Han Chinese
13	66	Male	Asian	Han Chinese
14	65	Male	Asian	Han Chinese
15	73	Male	Asian	Han Chinese
16	79	Male	Asian	Han Chinese
17	80	Male	Asian	Han Chinese
18	71	Male	Asian	Han Chinese
19	68	Male	Asian	Han Chinese
20	69	Male	Asian	Han Chinese

Table S4. The clinical information of 174 patients with BCa in STPH. Related to Figure 5.

Number	Age	Gender	Race	Ethnicity	Grade	DFStime(year)	DFSstate
1	66	Male	Asian	Han Chinese	High	1.666666667	0
2	81	Male	Asian	Han Chinese	High	1.333333333	0
3	77	Male	Asian	Han Chinese	High	0.916666667	0
4	61	Male	Asian	Han Chinese	High	0.583333333	0
5	58	Male	Asian	Han Chinese	Low	2	0
6	72	Male	Asian	Han Chinese	High	0.5	0
7	62	Female	Asian	Han Chinese	Low	2.083333333	0
8	82	Male	Asian	Han Chinese	High	1.083333333	0
9	65	Male	Asian	Han Chinese	High	2	0
10	82	Male	Asian	Han Chinese	High	1.666666667	0
11	61	Male	Asian	Han Chinese	High	1.5	0
12	66	Male	Asian	Han Chinese	High	1.666666667	0
13	63	Male	Asian	Han Chinese	High	0.333333333	1
14	86	Male	Asian	Han Chinese	Low	2	0
15	77	Female	Asian	Han Chinese	High	1	0
16	73	Male	Asian	Han Chinese	High	1.083333333	0
17	78	Female	Asian	Han Chinese	High	0.333333333	1
18	57	Female	Asian	Han Chinese	Low	1.916666667	0
19	72	Male	Asian	Han Chinese	High	0.333333333	1
20	61	Female	Asian	Han Chinese	Low	1.916666667	0
21	59	Female	Asian	Han Chinese	High	0.75	1
22	67	Male	Asian	Han Chinese	Low	1.916666667	0
23	87	Male	Asian	Han Chinese	High	0.333333333	1
24	64	Male	Asian	Han Chinese	High	1.75	0
25	71	Male	Asian	Han Chinese	High	1	1
26	87	Male	Asian	Han Chinese	High	0.75	1
27	70	Male	Asian	Han Chinese	High	0.25	1
28	68	Female	Asian	Han Chinese	High	1.166666667	0
29	79	Male	Asian	Han Chinese	High	1.166666667	0
30	50	Male	Asian	Han Chinese	High	0.916666667	0
31	64	Male	Asian	Han Chinese	High	0.833333333	0
32	81	Male	Asian	Han Chinese	Low	1.5	0
33	60	Male	Asian	Han Chinese	High	0.5	0
34	74	Male	Asian	Han Chinese	High	1.166666667	0
35	84	Female	Asian	Han Chinese	High	0.666666667	0
36	73	Male	Asian	Han Chinese	High	1.666666667	0
37	63	Female	Asian	Han Chinese	High	0.583333333	1
38	68	Male	Asian	Han Chinese	High	0.416666667	0
39	50	Female	Asian	Han Chinese	High	0.666666667	1

40	77	Female	Asian	Han Chinese	High	0.333333333	1
41	43	Female	Asian	Han Chinese	High	0.666666667	1
42	63	Male	Asian	Han Chinese	High	1.666666667	0
43	67	Male	Asian	Han Chinese	High	1	1
44	90	Male	Asian	Han Chinese	High	0.5	1
45	86	Male	Asian	Han Chinese	High	1	0
46	69	Male	Asian	Han Chinese	High	0.25	1
47	76	Male	Asian	Han Chinese	High	0.916666667	1
48	59	Male	Asian	Han Chinese	High	0.583333333	0
49	73	Male	Asian	Han Chinese	High	1.583333333	0
50	66	Male	Asian	Han Chinese	High	1.083333333	0
51	68	Female	Asian	Han Chinese	Low	1.583333333	0
52	68	Female	Asian	Han Chinese	Low	0.25	1
53	53	Male	Asian	Han Chinese	High	1	0
54	70	Male	Asian	Han Chinese	High	0.833333333	0
55	60	Male	Asian	Han Chinese	High	1.333333333	1
56	81	Male	Asian	Han Chinese	Low	0.75	1
57	78	Male	Asian	Han Chinese	High	0.083333333	1
58	83	Male	Asian	Han Chinese	High	0.666666667	1
59	66	Male	Asian	Han Chinese	High	0.333333333	1
60	63	Male	Asian	Han Chinese	High	1.75	0
61	87	Male	Asian	Han Chinese	High	0.5	1
62	70	Male	Asian	Han Chinese	High	2.75	0
63	65	Male	Asian	Han Chinese	High	1.666666667	0
64	70	Male	Asian	Han Chinese	High	1.416666667	0
65	70	Female	Asian	Han Chinese	High	0.916666667	0
66	48	Male	Asian	Han Chinese	Low	1.416666667	0
67	59	Male	Asian	Han Chinese	Low	1.416666667	0
68	72	Male	Asian	Han Chinese	High	0.666666667	0
69	86	Male	Asian	Han Chinese	High	0.916666667	0
70	71	Male	Asian	Han Chinese	Low	1.416666667	0
71	68	Female	Asian	Han Chinese	High	1.083333333	0
72	80	Male	Asian	Han Chinese	Low	1.416666667	0
73	75	Male	Asian	Han Chinese	High	1.75	0
74	74	Male	Asian	Han Chinese	High	1	0
75	56	Male	Asian	Han Chinese	High	1.583333333	0
76	64	Male	Asian	Han Chinese	Low	1.333333333	0
77	72	Female	Asian	Han Chinese	Low	1.333333333	0
78	72	Male	Asian	Han Chinese	High	0.833333333	0
79	94	Male	Asian	Han Chinese	High	0.75	0
80	57	Male	Asian	Han Chinese	High	2	0
81	84	Male	Asian	Han Chinese	High	0.5	0
82	67	Male	Asian	Han Chinese	High	1.416666667	0

83	74	Male	Asian	Han Chinese	High	1.25	0
84	77	Male	Asian	Han Chinese	High	1.25	0
85	50	Male	Asian	Han Chinese	Low	1.333333333	0
86	51	Male	Asian	Han Chinese	High	0.75	1
87	72	Male	Asian	Han Chinese	High	0.666666667	0
88	75	Male	Asian	Han Chinese	High	1.5	0
89	85	Male	Asian	Han Chinese	High	1.916666667	0
90	65	Male	Asian	Han Chinese	High	1.916666667	0
91	71	Male	Asian	Han Chinese	High	0.75	0
92	31	Female	Asian	Han Chinese	Low	1.25	0
93	68	Female	Asian	Han Chinese	High	1.25	0
94	69	Male	Asian	Han Chinese	High	1.5	0
95	83	Male	Asian	Han Chinese	High	0.333333333	1
96	52	Male	Asian	Han Chinese	Low	1.25	0
97	80	Male	Asian	Han Chinese	High	1.25	0
98	81	Male	Asian	Han Chinese	High	2	0
99	65	Male	Asian	Han Chinese	High	1.75	0
100	80	Male	Asian	Han Chinese	High	1.166666667	0
101	95	Female	Asian	Han Chinese	Low	1.166666667	0
102	71	Male	Asian	Han Chinese	High	2.083333333	0
103	71	Male	Asian	Han Chinese	High	1.166666667	0
104	82	Male	Asian	Han Chinese	High	1.083333333	0
105	90	Female	Asian	Han Chinese	High	0.416666667	1
106	70	Female	Asian	Han Chinese	Low	1	1
107	65	Male	Asian	Han Chinese	Low	0.583333333	1
108	87	Male	Asian	Han Chinese	High	1.25	0
109	70	Male	Asian	Han Chinese	High	0.166666667	1
110	69	Male	Asian	Han Chinese	High	0.666666667	1
111	28	Male	Asian	Han Chinese	High	0.75	0
112	61	Male	Asian	Han Chinese	High	2.083333333	0
113	51	Male	Asian	Han Chinese	Low	0.5	0
114	84	Female	Asian	Han Chinese	Low	1.083333333	0
115	68	Male	Asian	Han Chinese	Low	1.083333333	0
116	39	Male	Asian	Han Chinese	High	1.5	0
117	63	Male	Asian	Han Chinese	High	1.166666667	0
118	66	Male	Asian	Han Chinese	High	0.666666667	1
119	76	Male	Asian	Han Chinese	High	0.416666667	0
120	82	Male	Asian	Han Chinese	High	0.75	1
121	72	Female	Asian	Han Chinese	High	2.083333333	0
122	69	Male	Asian	Han Chinese	High	0.416666667	1
123	64	Male	Asian	Han Chinese	High	0.5	0
124	86	Male	Asian	Han Chinese	High	0.5	1
125	90	Male	Asian	Han Chinese	High	1.416666667	1

126	75	Male	Asian	Han Chinese	High	1.333333333	0
127	75	Male	Asian	Han Chinese	Low	1	0
128	66	Female	Asian	Han Chinese	Low	1	0
129	76	Male	Asian	Han Chinese	High	1.333333333	0
130	49	Male	Asian	Han Chinese	Low	1	0
131	57	Male	Asian	Han Chinese	High	1.5	0
132	72	Female	Asian	Han Chinese	High	1.416666667	1
133	46	Male	Asian	Han Chinese	High	1.5	0
134	71	Male	Asian	Han Chinese	Low	1	0
135	64	Male	Asian	Han Chinese	High	0.666666667	0
136	78	Male	Asian	Han Chinese	High	1.75	0
137	90	Female	Asian	Han Chinese	High	0.333333333	1
138	85	Male	Asian	Han Chinese	High	0.75	0
139	76	Male	Asian	Han Chinese	Low	0.916666667	0
140	45	Male	Asian	Han Chinese	High	1.166666667	1
141	55	Female	Asian	Han Chinese	Low	0.916666667	0
142	66	Male	Asian	Han Chinese	High	0.5	1
143	76	Male	Asian	Han Chinese	High	1.166666667	0
144	91	Female	Asian	Han Chinese	High	0.333333333	1
145	65	Male	Asian	Han Chinese	High	0.666666667	1
146	78	Male	Asian	Han Chinese	High	1	0
147	80	Male	Asian	Han Chinese	High	2	0
148	25	Female	Asian	Han Chinese	Low	0.833333333	0
149	49	Male	Asian	Han Chinese	High	0.833333333	0
150	46	Male	Asian	Han Chinese	Low	0.75	0
151	74	Male	Asian	Han Chinese	High	1.083333333	1
152	81	Male	Asian	Han Chinese	High	0.916666667	0
153	70	Male	Asian	Han Chinese	High	1.333333333	0
154	58	Male	Asian	Han Chinese	High	0.416666667	0
155	38	Female	Asian	Han Chinese	Low	0.75	0
156	68	Male	Asian	Han Chinese	High	1.75	0
157	51	Male	Asian	Han Chinese	High	1.666666667	0
158	72	Male	Asian	Han Chinese	High	1.416666667	0
159	90	Male	Asian	Han Chinese	High	0.583333333	0
160	64	Male	Asian	Han Chinese	High	1.416666667	0
161	83	Male	Asian	Han Chinese	High	0.583333333	0
162	73	Male	Asian	Han Chinese	High	1.333333333	0
163	57	Male	Asian	Han Chinese	High	0.75	0
164	70	Male	Asian	Han Chinese	High	1	0
165	65	Male	Asian	Han Chinese	High	1.666666667	0
166	75	Male	Asian	Han Chinese	High	0.5	1
167	63	Male	Asian	Han Chinese	High	1.75	0
168	57	Male	Asian	Han Chinese	High	1.333333333	0

169	88	Female	Asian	Han Chinese	High	0.666666667	1
170	82	Male	Asian	Han Chinese	High	1.916666667	0
171	74	Male	Asian	Han Chinese	High	0.25	1
172	67	Male	Asian	Han Chinese	Low	0.5	0
173	85	Male	Asian	Han Chinese	High	1	0
174	87	Male	Asian	Han Chinese	High	1.166666667	0
