# Supplementary material for: circKDM1A suppresses bladder cancer progression by sponging miR-889-3p/CPEB3 and stabilizing p53 mRNA
Source: iScience. 2024 Mar 29;27(4):109624. doi: 10.1016/j.isci.2024.109624 (PMC11022052; doi:10.1016/j.isci.2024.109624)

Figure 3J

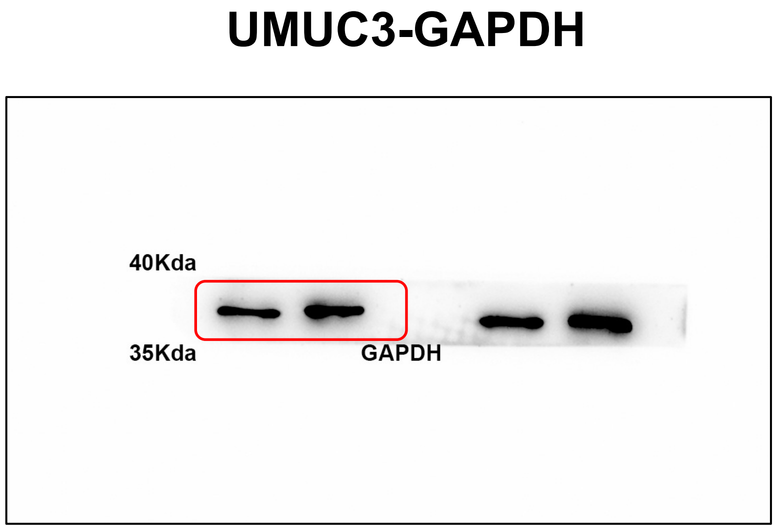
UMUC3-GAPDH
40Kda
35Kda
GAPDH

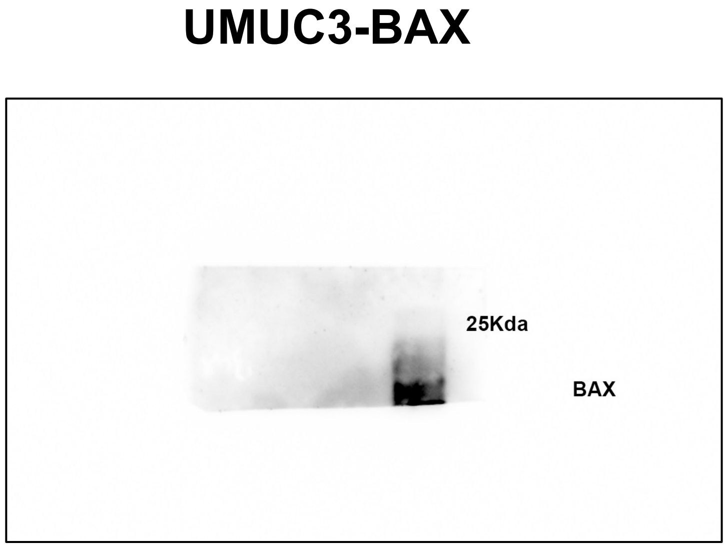
UMUC3-BAX
25Kda
BAX

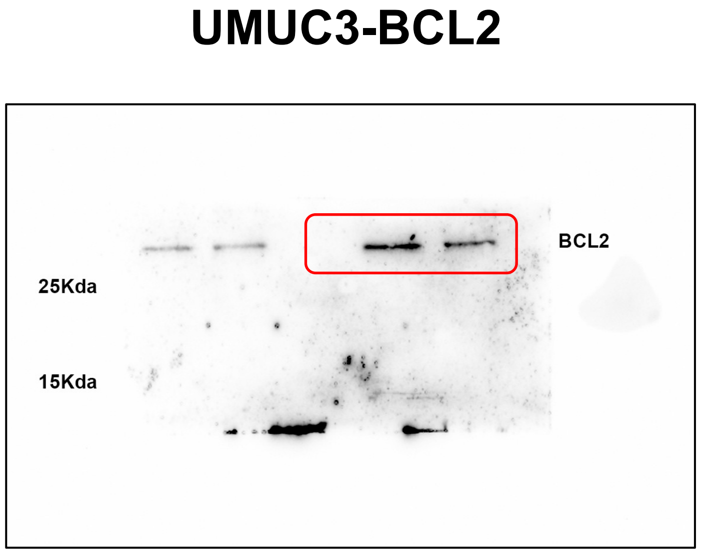
UMUC3-BCL2
BCL2
25Kda
15Kda

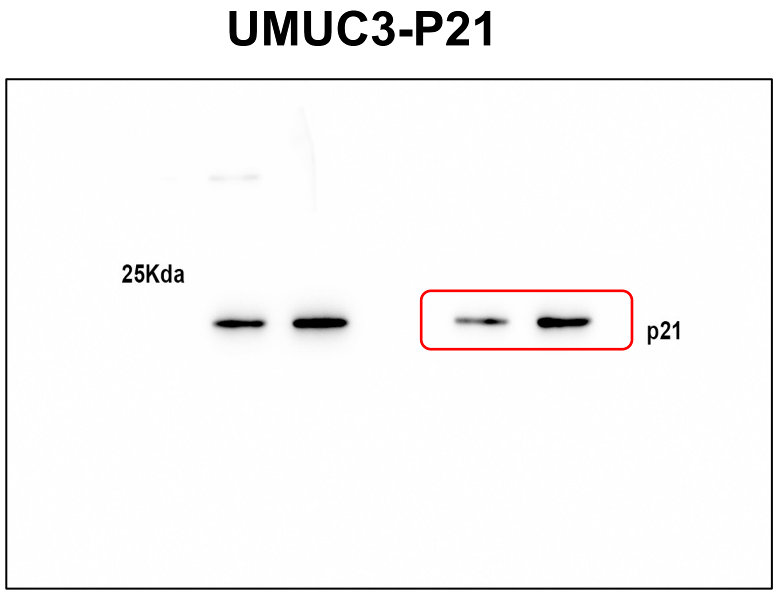
UMUC3-P21
25Kda
p21

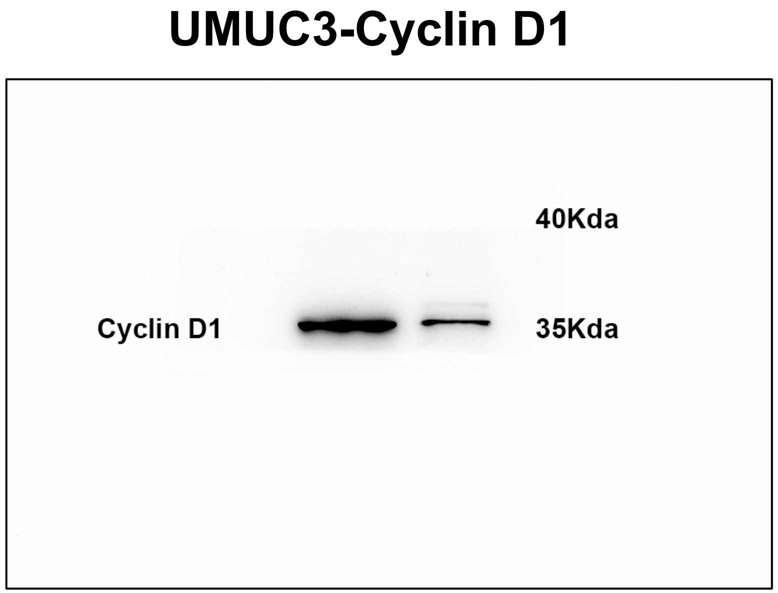
UMUC3-Cyclin D1
40Kda
Cyclin D1
35Kda

Figure 3J

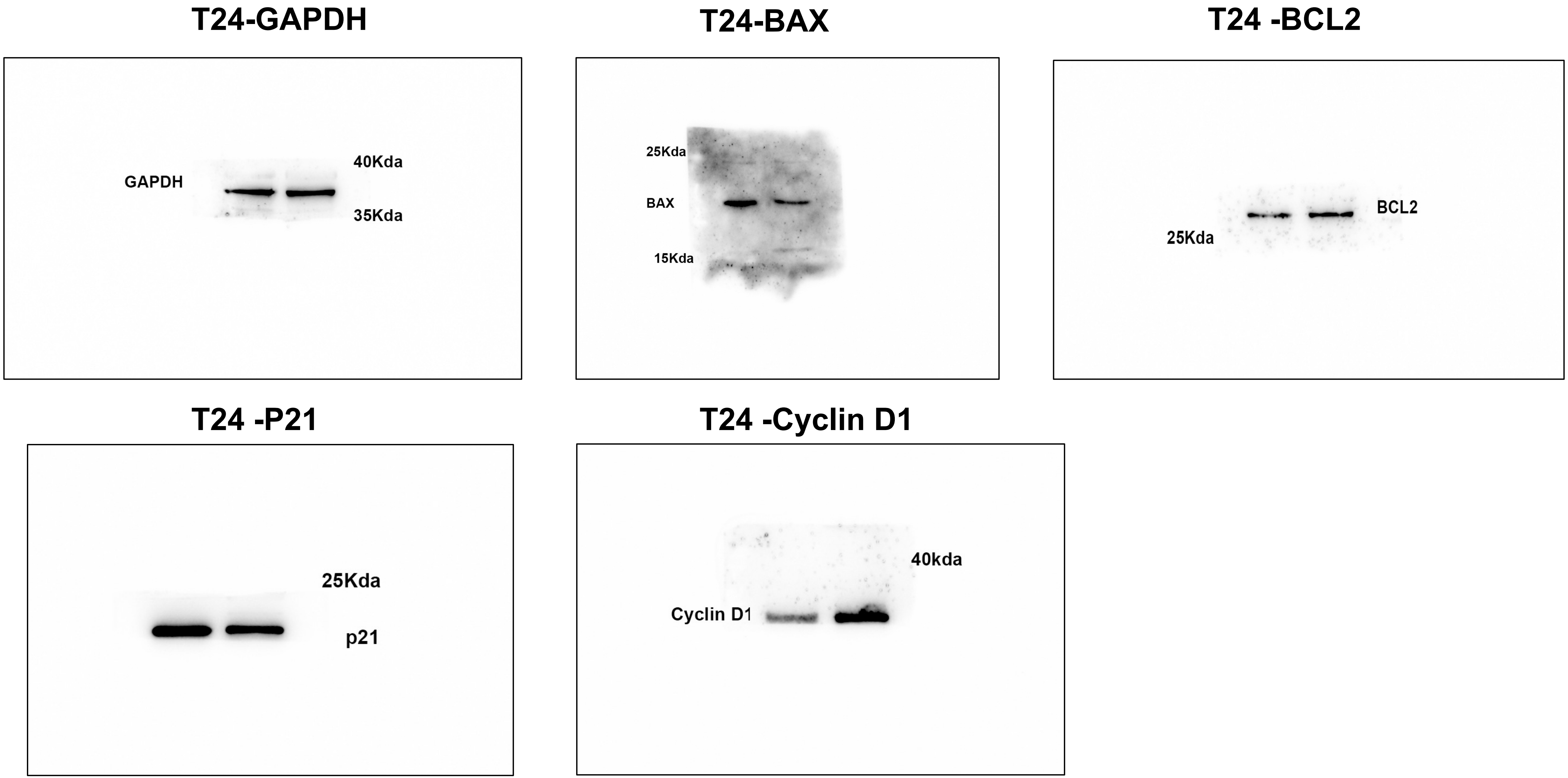
T24-GAPDH
GAPDH
40Kda
35Kda
T24-BAX
25Kda
BAX
15Kda
T24 -BCL2
BCL2
25Kda
T24 -P21
25Kda
p21
T24 -Cyclin D1
40kda
Cyclin D1

Figure 5N

UMUC3-CPEB3

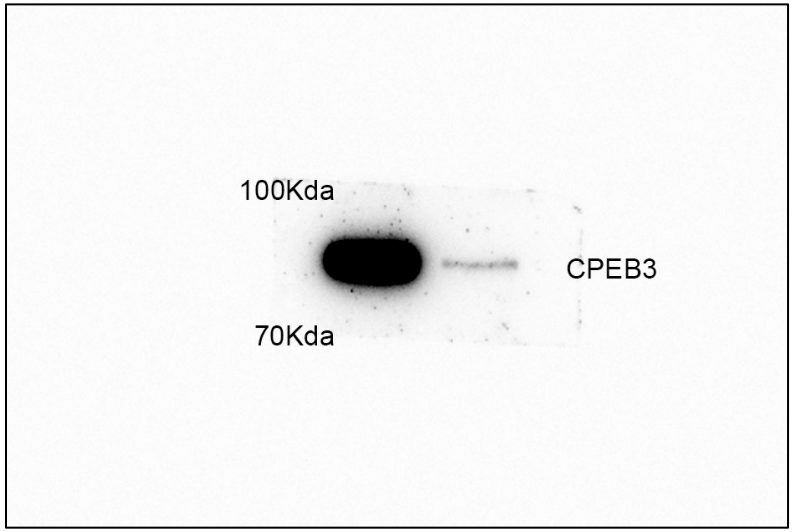

UMUC3-GAPDH

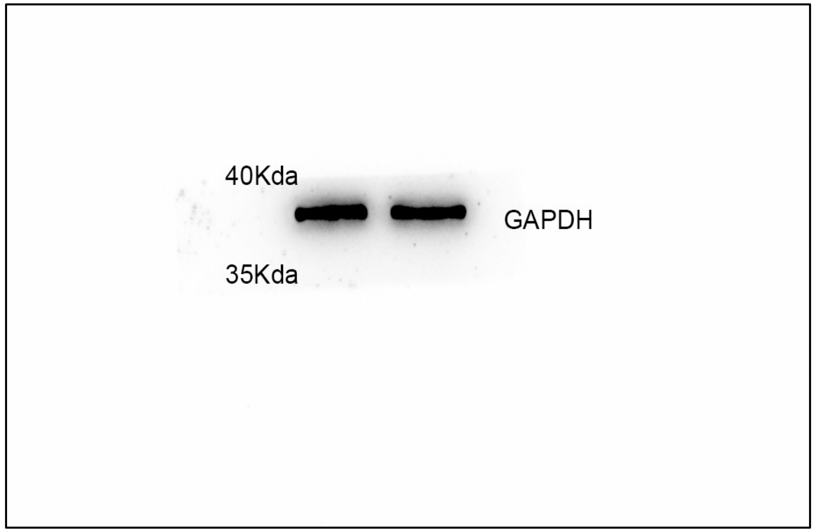

Figure 7G

p53

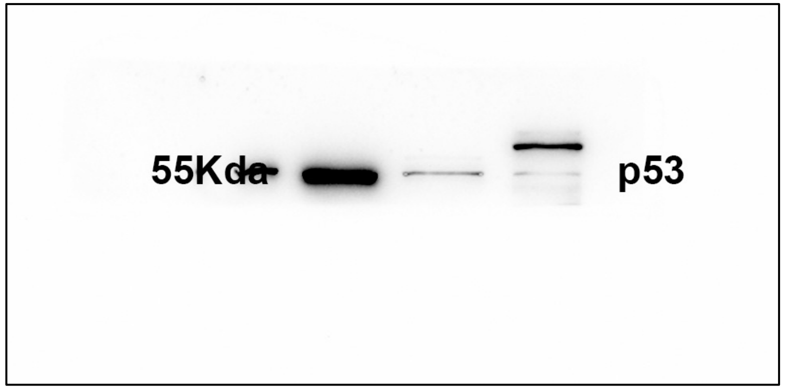

CPEB3

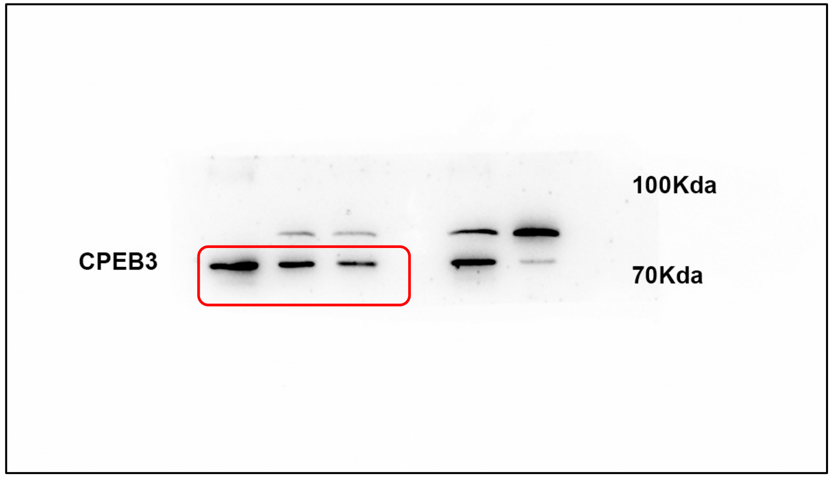

GAPDH

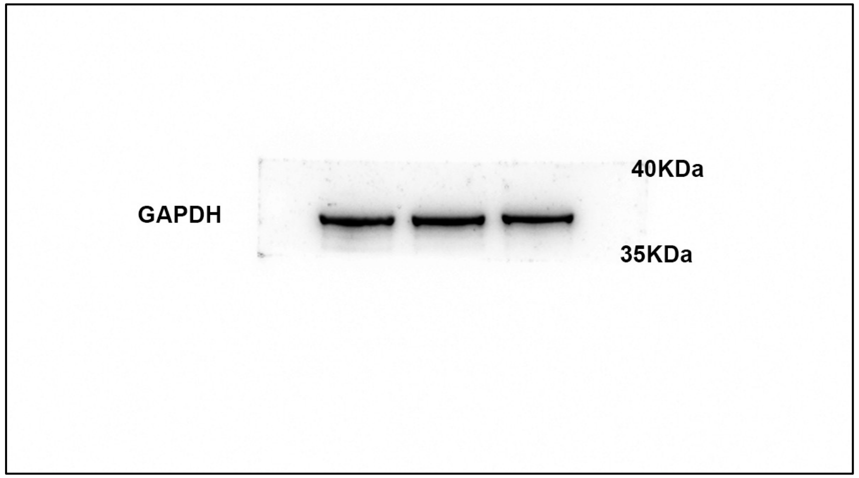

Supplement: Data S1. WB original data [file mmc2.pdf]
